# Single‐cell sequencing reveals the transcriptional alternations of 17β‐estradiol suppressing primordial follicle formation in neonatal mouse ovaries

**DOI:** 10.1111/cpr.13713

**Published:** 2024-07-10

**Authors:** Yutong Yan, Hui Zhang, Rui Xu, Linglin Luo, Lu Yin, Hao Wu, Yiqian Zhang, Chan Li, Sihai Lu, Yaju Tang, Xiaoe Zhao, Menghao Pan, Qiang Wei, Sha Peng, Baohua Ma

**Affiliations:** ^1^ College of Veterinary Medicine Northwest A&F University Yangling Shaanxi China; ^2^ Key Laboratory of Animal Biotechnology of the Ministry of Agriculture Northwest A&F University Yangling Shaanxi China

## Abstract

Estrogen has been implicated in multiple biological processes, but the variation underlying estrogen‐mediated primordial follicle (PF) formation remains unclear. Here, we show that 17β‐estradiol (E_2_) treatment of neonatal mice led to the inhibition of PF formation and cell proliferation. Single‐cell RNA sequencing (scRNA‐seq) revealed that E_2_ treatment caused significant changes in the transcriptome of oocytes and somatic cells. E_2_ treatment disrupted the synchronised development of oocytes, pre‐granulosa (PG) cells and stromal cells. Mechanistically, E_2_ treatment disrupted several signalling pathways critical to PF formation, especially down‐regulating the Kitl and Smad1/3/4/5/7 expression, reducing the frequency and number of cell communication. In addition, E_2_ treatment influenced key gene expression, mitochondrial function of oocytes, the recruitment and maintenance of PG cells, the cell proliferation of somatic cells, as well as disordered the ovarian microenvironment. This study not only revealed insights into the regulatory role of estrogen during PF formation, but also filled in knowledge of dramatic changes in perinatal hormones, which are critical for the physiological significance of understanding hormone changes and reproductive protection.

## Graphical abstract

The work first presented the transcriptional programmes of primordial follicle (PF) formation regulated by 17β‐estradiol in neonatal mouse ovaries, which not only revealed insights into the regulatory role of estrogen during PF formation, but also filled in knowledge of dramatic changes in perinatal hormones, which are critical for the physiological significance of understanding hormone changes and reproductive protection.

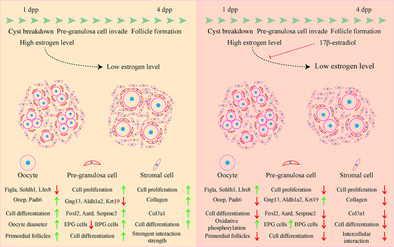

## 1 INTRODUCTION

Primordial follicle (PF) is the basic and non‐renewable unit of female mammals,^1^, ^2^ which is established pre‐ or perinatally. PF formation involves the cyst breakdown (CBD) of germ cells and invasion of pre‐granulosa (PG) cells and is an extremely complex biological process. The timing of this process varies among mammalian species; it occurs about 16 weeks postfertilization in human,^3^ while in mice, it begins at embryonic day 17.5 (E17.5) and completes about 4 dpp,^4^, ^5^, ^6^, ^7^ and the majority of PFs formation occurs perinatally,^8^ then the progressive and irreversible activation of PFs begins followed by pool formation. Specially, it is crucial for the synchronous development and cellular interaction between oocytes and ovarian somatic cells during PF formation.^4^, ^9^, ^10^

It is essential for oocyte survival and establishment with an appropriate quantity and normal function of PG cells.^11^, ^12^ In mice, two distinct pathways of PG cell differentiation support PF formation.^13^, ^14^ Bipotential pre‐granulosa (BPG) cells early express the transcription factor forkhead box L2 (*Foxl2*) to form the first wave of PFs, and Foxl2 is considered the most important agent for granulosa cell differentiation and maintenance;^12^, ^15^, ^16^ another population is epithelial pre‐granulosa (EPG) cells that express leucine‐rich repeat‐containing G‐protein‐coupled receptor 5 (*Lgr5*), a marker of EPG cells.^14^, ^17^, ^18^ Physiologically, EPG cells will eventually recruit and differentiate into BPG cells to form the second wave of PFs during ovarian development.^4^, ^13^

The oocyte‐specific expression of transcription factor, factor in the germline alpha (Figla), spermatogenesis and oogenesis specific basic helix‐loop‐helix 1 (Sohlh1), Sohlh2 and LIM homeobox 8 (Lhx8) also play essential roles in CBD and PF formation in mice and humans.^3^, ^5^ Compared with the extensively investigated follicles, ovarian non‐follicle cells have been rarely studied, and the understanding of their characteristics and functions remains insufficient.^19^ Especially stromal cells, considering a frontier,^20^ are also identified as the major cell population of non‐follicle cells from single‐cell RNA sequencing (scRNA‐seq) analysis.^21^, ^22^, ^23^ Moreover, follicles are surrounded by extracellular matrix (ECM) material within the ovarian stroma, which provides a supporting scaffold for the developing follicle and a reservoir for paracrine factors. Especially, ECM is vital for supporting the intercellular interactions and communication needed for follicle formation, development and migration within the ovary.^24^ During PF formation, multiply factors and signalling pathways are also implicated in the process, such as Notch, Kit/Kitl, transforming growth factor‐beta (TGF‐β) and estrogen.^25^, ^26^

Estrogen plays a vital role in ovarian development, and its main active form is E_2_.^27^ Historically, the role of estrogen in PF formation has become a hot topic in the field of reproduction,^8^, ^28^, ^29^, ^30^, ^31^, ^32^, ^33^, ^34^ which varies greatly in different species. Representatively, estrogen promotes CBD and PF formation in hamsters^31^ and baboons,^35^ while it is reversed in fetal bovine.^30^ Interestingly, human maternal estrogen has been maintained at a high level during PF formation.^36^ In the rodent, estrogen concentrations in the circulating perinatal serum level and ovaries peak in E17.5, but drop rapidly after birth.^8^, ^28^ Injection of E_2_ or synthetic estrogen analogues in newborn mouse results in a decrease in the number of PFs and the appearance of multiple oocyte follicle (MOF).^33^, ^37^, ^38^ In fetal or newborn mouse ovaries, E_2_ or estrogen analogues treatment inhibits the processes of CBD and PF formation.^33^ The specific agonist of estrogen receptor 1 (Esr1) or estrogen receptor 2 (Esr2) also can inhibit these processes,^29^ yet the knockout of *Esr1*, *Esr2* or both does not affect the PF formation,^39^, ^40^, ^41^ which suggests that estrogen may act through membrane receptor. Bovine serum albumin (BSA)‐E_2_ treats the newborn mouse ovaries and results in a reduction in the number of PFs,^29^ which suggests that estrogen regulates these processes through the extramembrane of estrogen receptor (G‐protein‐coupled estrogen receptor 1 [Gper1]). Taken together, previous studies have provided some phenotypes of estrogen action during PF formation, but the broad scope of estrogen action and complexity of regulation require further elucidation. Especially, these relevant researches focus on ovarian tissue and seriously ignore cell heterogeneity,^20^, ^42^ which limit the understanding of estrogen role during PF formation.

In recent years, scRNA‐seq technology has made it possible to identify specific cell subpopulations, which has revolutionised the dissection of cellular heterogeneity and cell fate transition from the single‐cell level.^43^, ^44^ Here, the present study aims to understand the transcriptional alternations by which E_2_ treatment inhibits PF formation. We daily administered super‐physiological dose of E_2_ in 1 dpp mice and performed transcriptomic analyses with scRNA‐seq in 4 dpp ovaries. We hypothesised that comparing gene expression signatures between control (CTRL) and E_2_‐treated ovaries would reveal previously uncharacterized features and thereby help elucidate the transcriptional alternations associated with ovarian suppression by E_2_, which would provide new insights into the role of estrogen during PF formation.

## 2 RESULTS

### 2.1 Neonatal mice injection of E_2_ inhibited PF formation and cell proliferation of somatic cells

**FIGURE 1:**
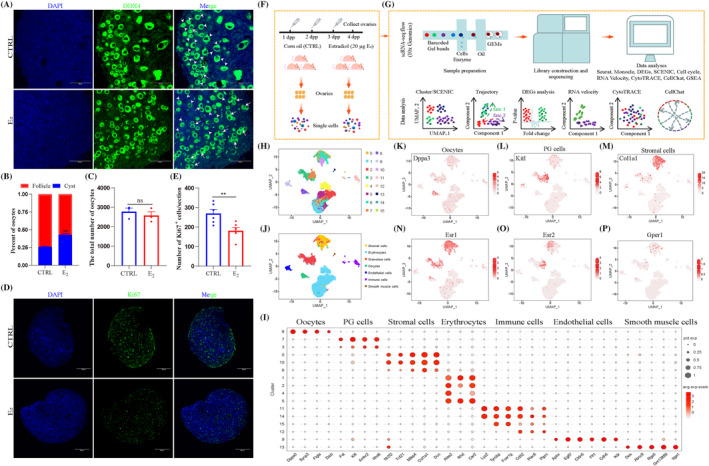
17β‐Estradiol (E_2_) treatment inhibited primordial follicle formation and analysed the responding cell types using single‐cell RNA sequencing (scRNA‐seq). (A) DDX4 and (2‐(4‐Amidinophenyl)‐6‐indolecarbamidine dihydrochloride) (DAPI) staining in 4 dpp ovaries between control (CTRL) and E_2_ groups. Oocytes and cell nuclei were marked with DDX4 and DAPI, respectively. Arrowhead: primordial follicle; arrow: cyst; scale bar: 50 μm. (B) The percentage of oocytes within cysts and follicles in CTRL and E_2_ groups. (C) The total number of oocytes in CTRL and E_2_ groups. (D, E) Staining and analysis of Ki67 in CTRL and E_2_ groups. Cell nuclei were stained with DAPI. Scale bar: 100 μm. (F, G) Schematic diagram of treatment, scRNA‐seq and data analyses. After treatment, ovaries were isolated and digested into single cell; after barcoding cells, performed with 10× Genomics platform and sequenced; data was analysed with Seurat, Monocle, different expression genes (DEGs), single‐cell regulatory network inference and clustering (SCENIC), Cell cycle, RNA velocity, CytoTRACE, CellChat and gene set enrichment analysis (GSEA). (H) Uniform manifold approximation and projection (UMAP) plots of ovarian cells based on 16 clusters in 4 dpp ovaries. (I) Dot plot of marker genes identified from ovarian cell types in each cluster. (J) The seven main ovarian cell types were identified on UMAP plots in 4 dpp ovaries. (K–M) Feature plots of representative marker genes for oocytes, pre‐granulosa (PG) cells and stromal cells, respectively. (N–P) Feature plots of *Esr1*, *Esr2* and *Gper1* for the seven main ovarian cell types, respectively. Data are shown as means ± SEM. All experiments were repeated at least three times (**p < 0.01). GEMs, gel beads in emulsions; ns, no significance.

To explore the role of estrogen during PF formation, neonatal mice were given a daily injection of 20 μg E_2_ in 1 dpp for 3 days and then the 4 dpp ovaries were isolated to analyse. Firstly, E_2_ treatment had no significant effect on the body weight of 4 dpp pups as well as ovarian area (Figure S1A–C). The staining of ovarian sections showed that E_2_ treatment inhibited PF formation and had a limited effect of oocyte number (Figures 1A and S1D–G). Whole ovarian follicle count analysis showed that E_2_ treatment decreased the proportion of PFs (CTRL: 74.24% ± 0.80%; E_2_: 57.25% ± 2.13%; Figure 1B), while had insignificant effect on the total number of oocytes (CTRL: 2770 ± 186; E_2_: 2570 ± 201; Figure 1C) and MVH expression (Figure S1H–J). E_2_ treatment had no significant effect on cell apoptosis (Figure S2A–D), but significantly decreased the capability of cell proliferation, presented the down‐regulation of *Ki67* and *Top2a* (Figure S2E,F), Proliferating cell nuclear antigen (PCNA) expression (Figure S2G,H), and also down‐regulated the number of Ki67 positive cells and Ki67 positive region (Figures 1D,E and S2I). Moreover, MOFs were presented in 21 dpp ovary after E_2_ treatment (Figure S2J), the incidence of MOFs was 100% (mice with MOFs/mice with examined: 8/8); MOFs were also presented in the adult ovary of the E_2_ group (mice with MOFs/mice with examined: 6/6) (Figure S2K), and the number of PFs was significantly decreased in both periods (Figure S2L,M). Taken together, these results indicated that E_2_ treatment disrupted PF formation and inhibited the proliferation of somatic cells.

### 2.2 The scRNA‐seq analyses identified seven main cell types in neonatal mouse ovaries

For characterising the transcriptome dynamics of ovarian cells affected by E_2_ treatment, the ovarian tissues were prepared into a single‐cell suspension; combined with barcoded gel beads, enzyme mixtures and oil; the gel beads in emulsions (GEMs) were generated for library construction and sequenced using scRNA‐seq (Figure 1F,G). The datasets were conducted for cell cluster, single‐cell regulatory network inference and clustering (SCENIC), trajectory, different expression genes (DEGs) analysis, RNA velocity, CytoTRACE, CellChat, cell cycle and gene set enrichment analysis (GSEA) (Figure 1G). After quality control of the data, 10,899 and 10,292 cells were left and used for subsequent analyses in the CTRL and E_2_ groups (Figure S3A,B), respectively. Parameters of sequencing data and scatter plots of the basic information are shown in Figure S3C,D, respectively.

According to uniform manifold approximation and projection (UMAP) in the two groups (Figure S4A), 16 cell clusters were generated (Figure 1H); the cell proportion is shown in Figure S4B. The number of up‐regulated genes in each cluster is shown in Figure S4C (Table S3), the top five up‐regulated genes are presented in Figure S4D, the correlated heatmap of each cluster is analysed in Figure S4E. According to the expression and distribution of characteristic genes in different cell types (Figure 1I), 16 cell clusters were identified into seven cell types (Figure 1J), which could be subdivided as follows: oocytes (cluster 8) with *Dppa3*, *Sycp3*, *Figla* and *Dazl* expression; PG cells (clusters 3 and 7) with *Fst*, *Kitl*, *Amhr2* and *Wnt6* expression; stromal cells (clusters 0, 6 and 10) with *Nr2f2*, *Tcf21*, *Mfap4*, *Col1a1* and *Dcn* expression; erythrocytes (clusters 1, 2, 4 and 5) with *Alas2*, *Rhd* and *Car2* expression; immune cells (clusters 11, 12, 14 and 15) with *Lyz2*, *Tyrobp*, *Fcer1g*, *Cd52*, *Plac8* and *Ptprc* expression; endothelial cells (cluster 9) with *Aplnr*, *Egfl7*, *Cldn5*, *Flt1*, *Cdh5* and *Kdr* expression; smooth muscle cells (cluster 13) with *Des*, *Abcc9*, *Rgs5*, *Gm13889* and *Itga1* expression, and the representative genes are shown in Figures 1K–M and S4F–I.

Moreover, *Esr1* was mainly presented in oocytes, PG cells and stromal cells (Figure 1N); *Esr2* was mainly presented in PG cells and stromal cells (Figure 1O), while *Gper1* was slightly expressed in the three cell types (Figure 1P). Based on the distribution of estrogen receptor, the transcriptome dynamics were mainly focused on oocytes, PG cells and stromal cells, which responded to E_2_ treatment. Considering E_2_ treatment decreased the capacity of cell proliferation (Figure 1D,E and S2E–H), the cell cycle was analysed in the CTRL and E_2_ groups. The expression of the cyclin genes in different cell cycles is shown in Figure S4J, the cell proportion in different cell cycles was in Figure S4K. E_2_ treatment altered the cell cycle of ovarian cells; specifically, E_2_ treatment down‐regulated the proportion of M phase (13.97% vs. 12.17%), G2 phase (24.99% vs. 14.79%), while up‐regulating the proportion of non‐cyclin phase (40.48% vs. 45.55%), G1 phase (8.67% vs. 15.23%) and S phase (11.89% vs. 12.26%). Collectively, seven cell types were identified in 4 dpp ovaries; oocytes, PG cells and stromal cells were the cell type that responded to E_2_ treatment; the capacity of cell proliferation was inhibited after E_2_ treatment.

### 2.3 E_2_ treatment impacted oocyte development

**FIGURE 2:**
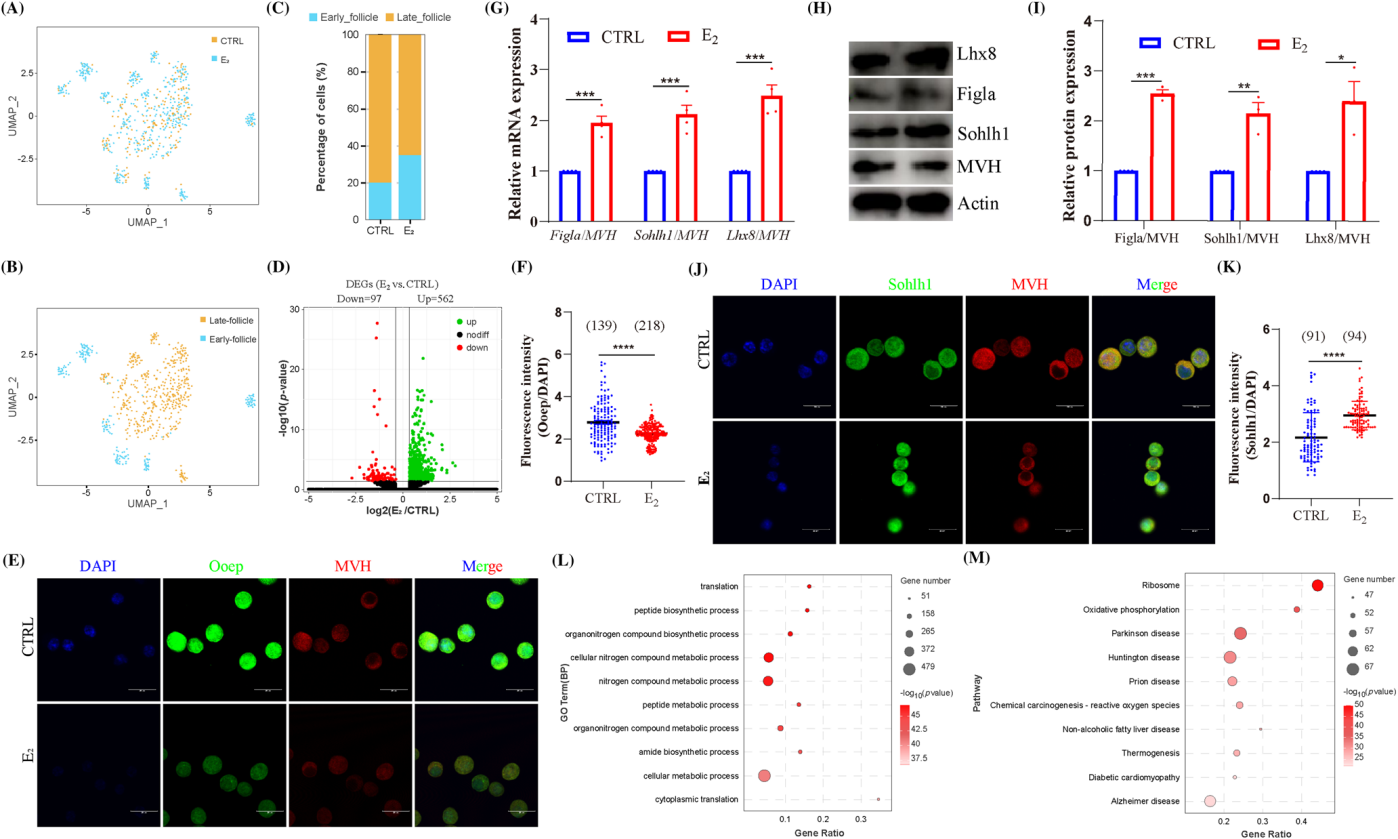
17β‐Estradiol (E_2_) treatment inhibited oocyte development and altered the expression of key genes. (A) Uniform manifold approximation and projection (UMAP) diagram of oocytes subpopulation from the control (CTRL) and E_2_ groups. (B, C) Oocytes were identified into early‐ and late‐follicles and their percentage, respectively. (D) Volcano plots of different expression genes (DEGs) in oocytes from CTRL and E_2_ groups. (E, F) The staining and analysis of *Ooep* in oocytes between CTRL and E_2_ groups, respectively. MVH marked oocytes, DAPI marked cell nuclei. Scale bar: 20 μm. (G) The mRNA expression of the key genes (*Figla*, *Sohlh1* and *Lhx8*) in 4 dpp ovaries. (H, I) The detection and analyses of protein (Figla, Sohlh1 and Lhx8) expression in ovaries. (J, K) The staining and analysis of Sohlh1 in oocytes between CTRL and E_2_ groups. MVH marked oocytes, DAPI marked cell nuclei. Scale bar: 20 μm. (L) Top 10 GO term (biological processes) of DEGs in oocytes from CTRL and E_2_ groups. (M) Top 10 pathways of the DEGs in oocytes. Data are shown as means ± SEM. All experiments were repeated at least three times (*p < 0.05, **p < 0.01, ***p < 0.001 and ****p < 0.0001).

For analysing the heterogeneity of oocytes after E_2_ treatment, oocytes were extracted (Figure 2A) and assigned into seven clusters (Figure S5A) with the UMAP projection; the top 10 genes are shown in Figure S5B. The typical marker genes (Figure S5C) were used to assign oocytes into different stages from previous reports.^45^, ^46^, ^47^ Briefly, *Stra8*, *Prdm9*, *Meioc*, *Hspb11*, *M1ap* and *Pigp* were labelled as pre‐PF formation stage; *Figla*, *Lhx8*, *Nobox*, *Sohlh1*, *Eif4a1*, *G3bp2*, *Acat1*, *Ldhb*, *Dppa3* and *Gdpd1* were marked as early‐PF formation stage; *Ooep*, *Ybx2*, *Padi6*, *Gm15389*, *Mvp* and *Nlrp5* were marked as late‐PF formation stage. Herein, these specific genes of the pre‐PF formation stage were expressed in a low level (Figure S5C), so oocytes were divided into early‐follicle (clusters 1–5) and late‐follicle (clusters 0 and 6) stages (Figure 2B). Comparing with the CTRL group, the proportion of early‐follicle was increased (20% vs. 34.85%), while the proportion of late‐follicle (80% vs. 65.15%) was decreased after E_2_ treatment (Figure 2C).

DEGs analysis of oocytes showed that there were 97 down‐ and 562 up‐regulated genes after E_2_ treatment (Figure 2D; Table S4), which were supported by *Fos* and *Kit* expression (Figure S5D–G). In order to explore the alterations, oocytes were collected after digesting the ovary tissues (Figure S5H), and the diameter of oocyte was significantly decreased after E_2_ treatment (CTRL: 17.32 ± 0.044; E_2_: 16.14 ± 0.042; Figure S5I). The *Ooep* expression was significantly decreased from DEGs analysis (Figure S5J), and immunofluorescence (IF) staining further supported the down‐regulation (Figure 2E,F). DEGs of oocytes showed that *Figla*, *Sohlh1* and *Lhx8* were significantly up‐regulated in the E_2_ group (Figure S5K–M) and were further supported in Figure 2G–K.

Gene Ontology (Go) terms (biological processes [BP]) (Figure 2L) demonstrated that 659 DEGs were mainly related to ‘translation,’ ‘peptide biosynthetic process,’ ‘organonitrogen compound biosynthetic process,’ ‘cellular nitrogen compound metabolic process,’ and were related to ‘ribosome,’ ‘oxidative phosphorylation’ pathways (Figure 2M). Furthermore, DEGs analysis was performed at the two identified stages of oocytes after E_2_ treatment. For oocytes in the early‐follicle stage, there were 1058 down‐ and 214 up‐regulated DEGs (Figure S6A; Table S14), while E_2_ treatment significantly increased 903 genes and decreased 322 genes in oocytes in the late‐follicle stage (Figure S6D; Table S15). Enrichment analysis of DEGs was performed in the early‐follicle stage (Figure S6B) and late‐follicle stage (Figure S6E), and Kyoto Encyclopedia of Genes and Genomes (KEGG) analysis was performed in the two stages (Figure S6C,F). Moreover, 248 DEGs were shared in early‐ and late‐follicle stages (Figure S6G), Go terms (BP) were mainly related with ‘organonitrogen compound metabolic process,’ ‘peptide metabolic process’ and ‘translation’ (Figure S6H), and the relative pathways were ‘ribosome,’ ‘oxidative phosphorylation’ (Figure S6I). Collectively, the development of oocytes was delayed after E_2_ treatment, and oxidative phosphorylation signalling was significantly enriched in oocytes.

### 2.4 E_2_ treatment affected the molecular composition of oocyte mitochondria and enhanced mitochondrial activity

**FIGURE 3:**
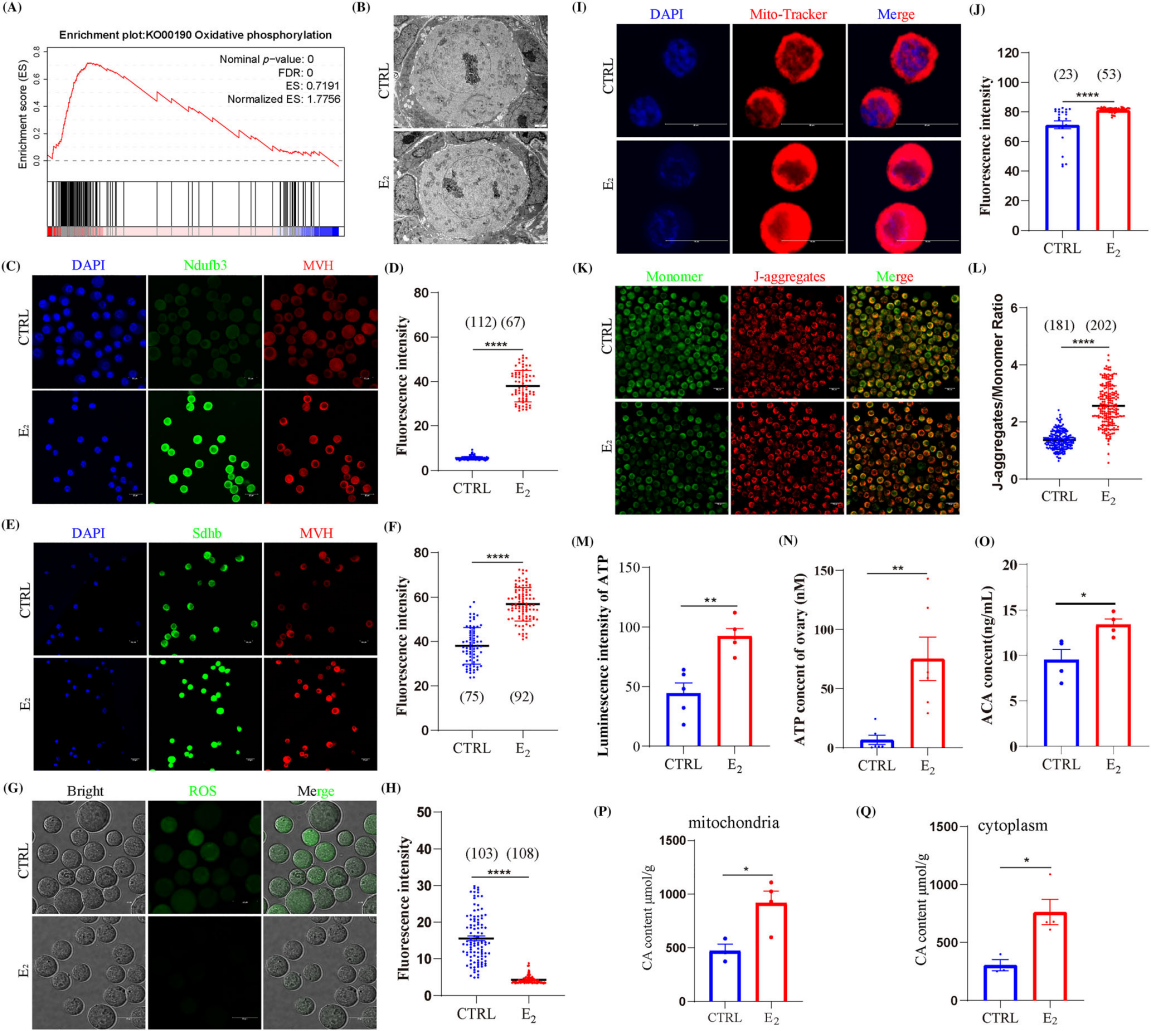
17β‐Estradiol (E_2_) treatment altered mitochondrial molecular composition and activity. (A) The gene set enrichment analysis of the oxidative phosphorylation pathway in oocytes between control (CTRL) and E_2_ groups. (B) Transmission electron microscopy observation of oocyte mitochondria in CTRL and E_2_ groups. (C–F) The staining and analyses of key genes (Ndufb3 and Sdhb) in oocytes related to ETC, respectively. MVH marked oocytes, DAPI marked cell nuclei. Scale bar: 20 μm. (G, H) The detection and analysis of ROS level in oocytes between CTRL and E_2_ groups. Scale bar: 20 μm. (I, J) The detection and analysis of Mito‐Tracker in oocytes between CTRL and E_2_ groups. Scale bar: 20 μm. (K, L) The detection and analysis of membrane potential level in oocytes between CTRL and E_2_ groups. Scale bar: 20 μm. (M, N) ATP content of oocytes and ovaries in CTRL and E_2_ groups, respectively. (O) Acetyl coenzyme A (ACA) content of ovaries in CTRL and E_2_ groups. (P, Q) Mitochondria and cytoplasm of citric acid (CA) content in CTRL and E_2_ ovaries. Data are shown as means ± SEM. All experiments were repeated at least three times (*p < 0.05, **p < 0.01 and ****p < 0.0001).

The DEGs analysis of oocytes mainly focused on the oxidative phosphorylation pathway, which was enhanced after E_2_ treatment from GSEA analysis (Figure 3A). Transmission electron microscopy (TEM) observation of the oocyte showed that mitochondria were intact and had no significant difference in membrane density and ridge after E_2_ treatment (Figure 3B). IF staining showed the significant up‐regulation of Ndufb3 (Figure 3C,D) and Sdhb (Figure 3E,F) in oocytes from E_2_‐treatment ovaries. Dichlorofluorescein staining showed that the Reactive oxygen species (ROS) level of oocytes from E_2_‐treated mice was significantly lower than that of the CTRL group (Figure 3G,H). The Mito‐Tracker staining showed that the number of active mitochondria were significantly increased in oocytes from E_2_‐treated ovaries (Figure 3I,J). The JC‐1 staining showed that mitochondrial membrane potential was increased after E_2_ treatment (Figure 3K,L). The ATP content was increased in oocytes (Figure 3M) and ovaries (Figure 3N). Moreover, the level of acetyl coenzyme A (ACA) and citric acid (CA) can reflect the tricarboxylic acid cycle flux and ETC activities.^48^ The level of ACA increased after E_2_ treatment (Figure 3O), and the CA content of mitochondria and cytoplasm were significantly higher in E_2_‐treated ovaries (Figure 3P,Q). Altogether, the molecular composition of mitochondria and mitochondrial activity were enhanced in oocytes after E_2_ treatment.

### 2.5 E_2_ treatment disordered cell fate of oocytes by single cell trajectory

**FIGURE 4:**
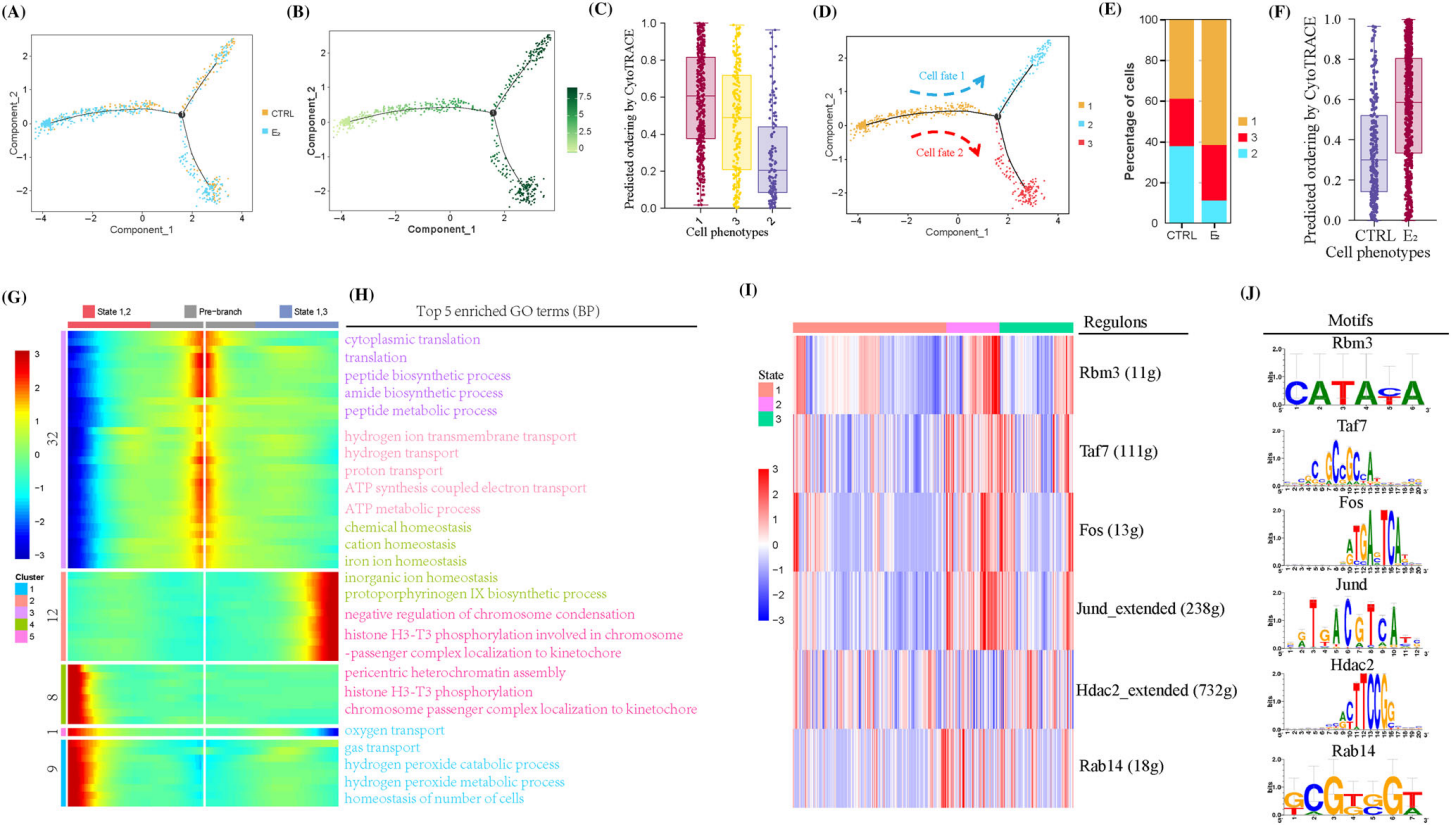
17β‐Estradiol (E_2_) treatment disordered the cell fates of oocytes. (A) The developmental trajectory of oocytes between the control (CTRL) and E_2_ groups in pseudotime. (B) The pseudotime information of oocytes in the CTRL and E_2_ groups. (C) Predicted ordering by CytoTRACE in three states of pseudotime. (D) Two cell fates of oocytes in pseutotime. (E) Percentage of oocytes in three cell states between CTRL and E_2_ groups. (F) Predicted ordering by CytoTRACE analysis in CTRL and E_2_ groups. (G) Heatmap of the gene expression programmes in oocytes from two cell fates. (H) Top 5 enriched GO terms (biological processes) of each gene set. (I) Heatmap of regulons activity in oocytes analysed by single‐cell regulatory network inference and clustering analysis in three cell states. (J) The representative motif of regulation.

For further dissection of the fate transition of oocytes after E_2_ treatment, the pseudotime trajectory of oocytes was established into three states (Figures 4A and S7A), the value of pseudotime is shown in Figure 4B. *Sycp3* and *Ppia* were highly expressed in the meiotic stage, while *Figla* and *Eif4a1* were actively expressed in early‐follicle stage. Along with cell trajectory, *Sycp3*, *Ppia*, *Figla* and *Eif4a1* were actively expressed at state1 (Figure S7B), so state1 may be the early stage. In order to prove the conjecture, oocytes were analysed with CytoTRACE (Figure S7C–E), the predicted ordering by CytoTRACE was higher at state1 than state2 and state3 (Figure 4C), so both further indicated that state1 was the origin site of differentiation, which can be differentiated into state2 and state3 (Figure 4D). Comparing with the CTRL group, the proportion of oocytes in state1 increased (39.05% vs. 61.65%) while decreasing in state2 (37.62% vs. 10.93%), and there was a limited change in state3 (23.33% vs. 27.42%) in the E_2_ group (Figure 4E). Compared with the predicted ordering by CytoTRACE in CTRL and E_2_ groups (Figure 4F), it is implied that PF formation was impeded for higher predicted ordering after E_2_ treatment.

The representative DEGs of two cell fates are shown in Figure S7F. For investigating the inherent mechanism, gene expression patterns at two branches were performed by cell fate (Figure 4G). Five gene sets and two cell fates were generated from 62 genes (Table S11); top five GO terms (BP) are shown in Figure 4H. Interestingly, the relative genes of ribosome subunits were gradually decreased from state3 to state2, while the mitochondrial genes encoded NADH dehydrogenase (*mt‐Nd1/2/3/4/4L/5*), cytochrome c oxidase (*mt‐Co1/2/3* and *mt‐Cytb*) or ATP synthase (*mt‐Atp6/8*) increased at state3 and decreased at state2. Moreover, the three states were performed with SCENIC analysis, which identified *Rbm3*, *Taf7*, *Fos*, *Jund*, *Hdac2* and *Rab14* as transcriptional regulators during this process. The regulon activity was shown according to three cell states (Figure 4I) and two groups (Figure S7G), and the representative motifs are shown in Figure 4J. Considering the down‐expression of *Fos* and *Jund* in the E_2_ group (Figures 4I and S7G), they may be the key regulons for responding to E_2_ treatment.

### 2.6 E_2_ treatment impacted the recruitment and maintenance of pre‐granulosa cells

**FIGURE 5:**
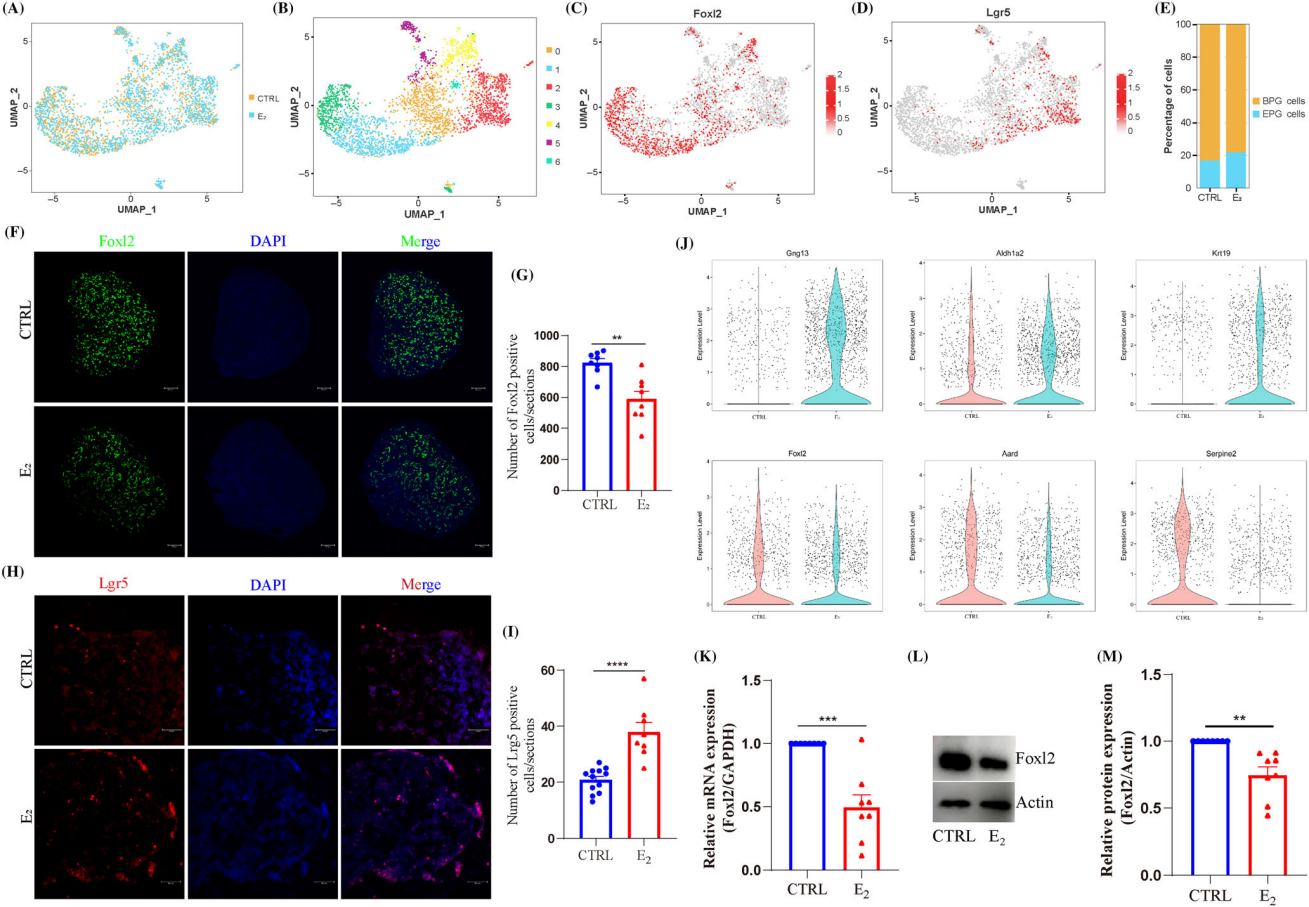
17β‐Estradiol (E_2_) treatment impacted the recruitment and maintenance of pre‐granulosa cells. (A, B) Clustering of pre‐granulosa (PG) cells with uniform manifold approximation and projection (UMAP) in the control (CTRL) and E_2_ groups, based on sample groups and seven clusters, respectively. (C, D) Expression and distribution of *Foxl2* and *Lgr5* in PG cells between CTRL and E_2_ groups, respectively. (E) The percentage of two types of PG cells in the CTRL and E_2_ groups. (F–I) The detection and analyses of two types of PG cells in CTRL and E_2_ groups. Fxol2 marked bipotential pre‐granulosa (BPG) cells, Lgr5 marked epithelial pre‐granulosa (EPG) cells and DAPI marked cell nuclei. Scale bar: 50 μm. (J) The representative characteristic DEGs (*Gng13*, *Aldh1a2*, *Krt19*, *Foxl2*, *Aard* and *Serpine2*) of PG cells in CTRL and E_2_ groups. (K–M) The detection and analysis of Foxl2 expression in 4 dpp ovaries between CTRL and E_2_ groups. Data are shown as means ± SEM. All experiments were repeated at least three times (**p < 0.01, ***p < 0.001, and ****p < 0.0001).

PG cells were extracted (Figure 5A) and subdivided into seven clusters with the subpopulation analysis (Figure 5B), and the up‐regulated genes were analysed (Table S16). According to the characteristic genes of EPG and BPG cells (Figure S8A) and the top 10 up‐regulated genes in each cluster (Figure S8B), cluster 2 belonged to EPG cells, which actively expressed *Lgr5*, *Gng13*, *Krt19*, *Lhx9*, *Apoc1*, *Folr1*, *Gpc3*, *Cst8*, *Bace2* and *Hmcn1*; clusters 0, 1, 3, 4, 5 and 6 were BPG cells, which were actively expressed *Foxl2*, *Hsd3b1*, *Aard*, *Akr1c14*, *Akr1cl*, *Cfh*, *Hmgcs2*, *Mgp* and *Col18a1*; the top 10 up‐regulated genes in BPG and EPG cells are shown in Figure S8C. PG cells expressed *Amhr2* (Figure S8D), and the representative characteristic genes of BPG and EPG cells are shown in Figure 5C,D. Compared with the CTRL group from classification (Figure 5E), the proportion of EPG cells was higher in the E_2_ group (16.63% vs. 21.59%), while the proportion of BPG cells was lower in the E_2_ group (83.37% vs. 78.41%). Moreover, the number of Foxl2 positive cells in the E_2_ group decreased (Figure 5F,G), while the number of Lgr5 positive cells increased (Figure 5H,I), which suggested that the recruitment of EPG cells into BPG cells was disordered after E_2_ treatment.

E_2_ treatment resulted in 2195 DEGs in PG cells (Figure S8E; Table S5). GO terms (BP) (Figure S8F) mainly focused on ‘cellular metabolic process,’ ‘metabolic process,’ ‘organic substance metabolic process,’ ‘primary metabolic process’ and ‘cellular component organisation or biogenesis’; KEGG analysis (Figure S8G) mainly focused on ‘ribosome’ and ‘oxidative phosphorylation.’ In Figure 5J, E_2_ treatment down‐regulated the expression of characteristic genes for BPG cells (*Foxl2*, *Aard* and *Serpine2*) and up‐regulated the specific genes for EPG cells (*Gng13*, *Aldh1a2* and *Krt19*). Physiologically, Foxl2 expression gradually increased after birth (Figure S9A,B). Comparing with the CTRL group, Foxl2 expression was significantly decreased in 4 dpp ovaries after E_2_ treatment (Figure 5K–M) and even to 21 dpp ovaries (Figure S9C,D). Moreover, *Nr5a2* and *Krt79* were down‐regulated in the E_2_ group (Figure S9E–H); and further supported the alterations of PG cells. Altogether, the maintenance of PG cells was disturbed after E_2_ treatment.

Moreover, DEGs were further analysed in the two types of PG cells. In EPG cells, E_2_ treatment significantly up‐regulated 1298 genes and down‐regulated 1018 genes (Figure S9I; Table S17), and GO analysis (BP) (Figure S9J) and KEGG analysis (Figure S9K) were analysed with these DEGs. For BPG cells, 1324 genes were down‐regulated and 834 genes were up‐regulated (Figure S9L; Table S18), the GO terms (BP) and KEGG analyses are shown in Figure S9M,N, respectively. Moreover, 858 DEGs were shared in EPG and BPG cells (Figure S9O) and mainly related to ‘organonitrogen compound metabolic process,’ ‘organonitrogen compound biosynthetic process’ and ‘cellular metabolic process’ (Figure S9P). Other unique DEGs involved in GO terms (BP) are shown in Figure S9Q,R.

### 2.7 E_2_ treatment inhibited the proliferation and disordered cell fate of pre‐granulosa cells

**FIGURE 6:**
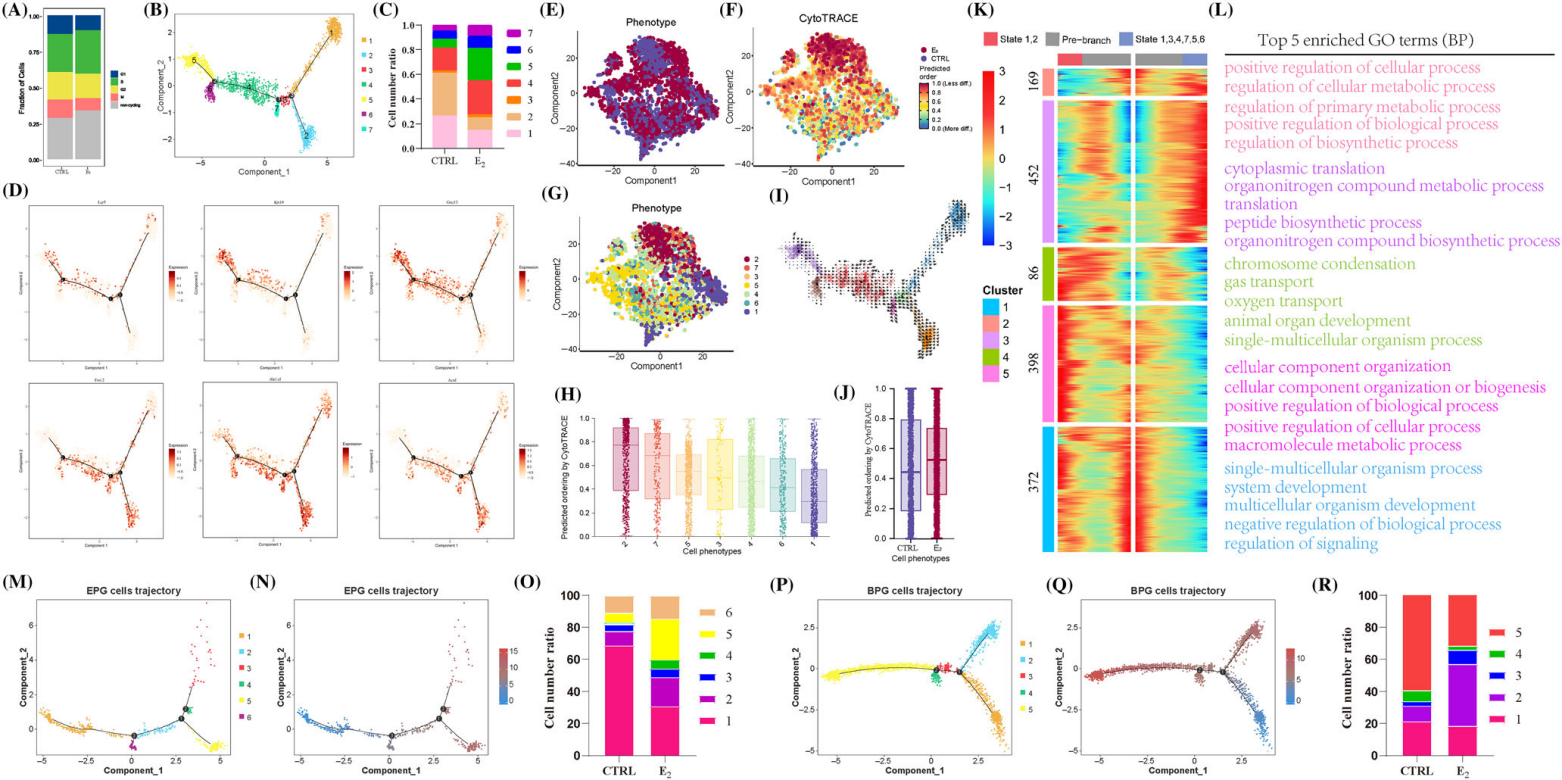
17β‐Estradiol (E_2_) treatment inhibited cell proliferation and disordered cell fates in pre‐granulosa (PG) cells. (A) The percentage of cell cycle phase in PG cells between control (CTRL) and E_2_ groups. (B) Uniform manifold approximation and projection (UMAP) plots of PG cells based on seven cell states in pseudotime. (C) Cell number ratio of seven cell states in PG cells. (D) The characteristic gene expression of epithelial pre‐granulosa (EPG) (*Lgr5*, *Krt19* and *Gng13*) and bipotential pre‐granulosa (BPG) (*Foxl2*, *Akr1cl* and *Aard*) cells in PG cells. (E–H) CytoTRACE analyses of PG cells based on samples and cell states. (I) RNA velocity analysis of PG cells in the CTRL and E_2_ groups. (J) The predicted ordering by CytoTRACE analysis in the CTRL and E_2_ groups. (K) Pseudotime ordered heatmap of five different expression genes sets between two cell fates at branch point three in CTRL and E_2_ groups. (L) Top 5 enriched GO terms (BP) of each gene set. (M, P) UMAP plots of EPG and BPG cells in pseudotime based on cell states, respectively. (N, Q) The pseudotime information of EPG and BPG cells, respectively. (O, R) Cell number ration of EPG and BPG cells based on cell fates, respectively. BP, biological processes.

The cell cycle analysis was performed in PG cells. The heatmap of different cell cycle genes is shown in Figure S10A, and the up‐regulated genes related to cell cycle are presented with a bubble map (Figure S10B). Results showed that E_2_ treatment inhibited the proliferation of PG cells (Figure 6A), presently, the proportion of cells in G1 phase (12.61% vs. 10.19%), G2 phase (19.23% vs. 17.04%) and M phase (12.69% vs. 8.45%) were decreased, and the up‐proportion in S phase (26.56% vs. 30.23%) and non‐cycling phase (28.91% vs. 34.09%). Moreover, DEGs analysis showed that *Mki67*, *Cdkn1b* and *Ccnd1* expression were significantly decreased in PG cells after E_2_ treatment (Figure S10C).

Subsequently, PG cells were further analysed with Monocle (Figure 6B), and the value of pseudotime is shown in Figure S10D. Monocle analysis showed that PG cells were divided into seven states (Figures 6B and S10E), the cell number ration was analysed in Figure 6C. The expression of characteristic genes in BPG and EPG cells is shown in Figure 6D. Representatively, *Lgr5* is mainly expressed in state5 while *Foxl2* is actively expressed in state1 and state2. CytoTRACE analysis was performed into PG cells and seven states (Figure 6E–H); the RNA velocity analysis was performed into Monocle and showed that state5 was differentiated into state2 (Figure 6I), which enhanced EPG cells differentiated into BPG cells in single‐cell level. Compared with the CTRL group, the percentage of state5 increased after E_2_ treatment (7.25% vs. 26.19%). Moreover, the predicted ordering of PG cells was increased in the E_2_ group (Figure 6J). According to monocle analysis, five gene sets were generated according to cell fates at branch 3 (Figure 6K; Table S12), top five enriched GO terms (BP) are shown in Figure 6L.

For reconstructing the pseudotime trajectory of PG cells, we performed the monocle analysis on EPG cells (Figure 6M,N) and BPG cells (Figure 6P,Q), and the cell number ratio is shown in Figure 6O,R, the representative DEGs from pseudotime were presented in Figure S10F,G. Results showed that EPG cells were divided into six states (Figure 6M); cell state1 mainly contained EPG cells in CTRL group (68.72% vs. 30.79%), and cell state2, 5 and 6 mainly contained EPG cells in the E_2_ group (58.10% vs. 25.59%). Next, we compared the gene expression profiles of EPG cells and observed six gene sets (Figure S11A; Table S19). The top five enriched GO terms (BP) are shown in Figure S11B. The pseudotime analysis showed that BPG cells were divided into five states (Figure 6P), state5 has the largest proportion of cells in the CTRL group (59.07% vs. 31.49%), while state2 has the largest proportion in E_2_ group (9.74% vs. 38.56%). Five gene sets showed different expression profiles (Figure S11C; Table S20); the top five enriched GO terms (BP) are shown in Figure S11D. Collectively, E_2_ treatment decreased the capacity for cell proliferation and disordered cell fates in PG cells as well as BPG and EPG cells.

### 2.8 E_2_ treatment impacted the development and disordered cell fate of stromal cells

**FIGURE 7:**
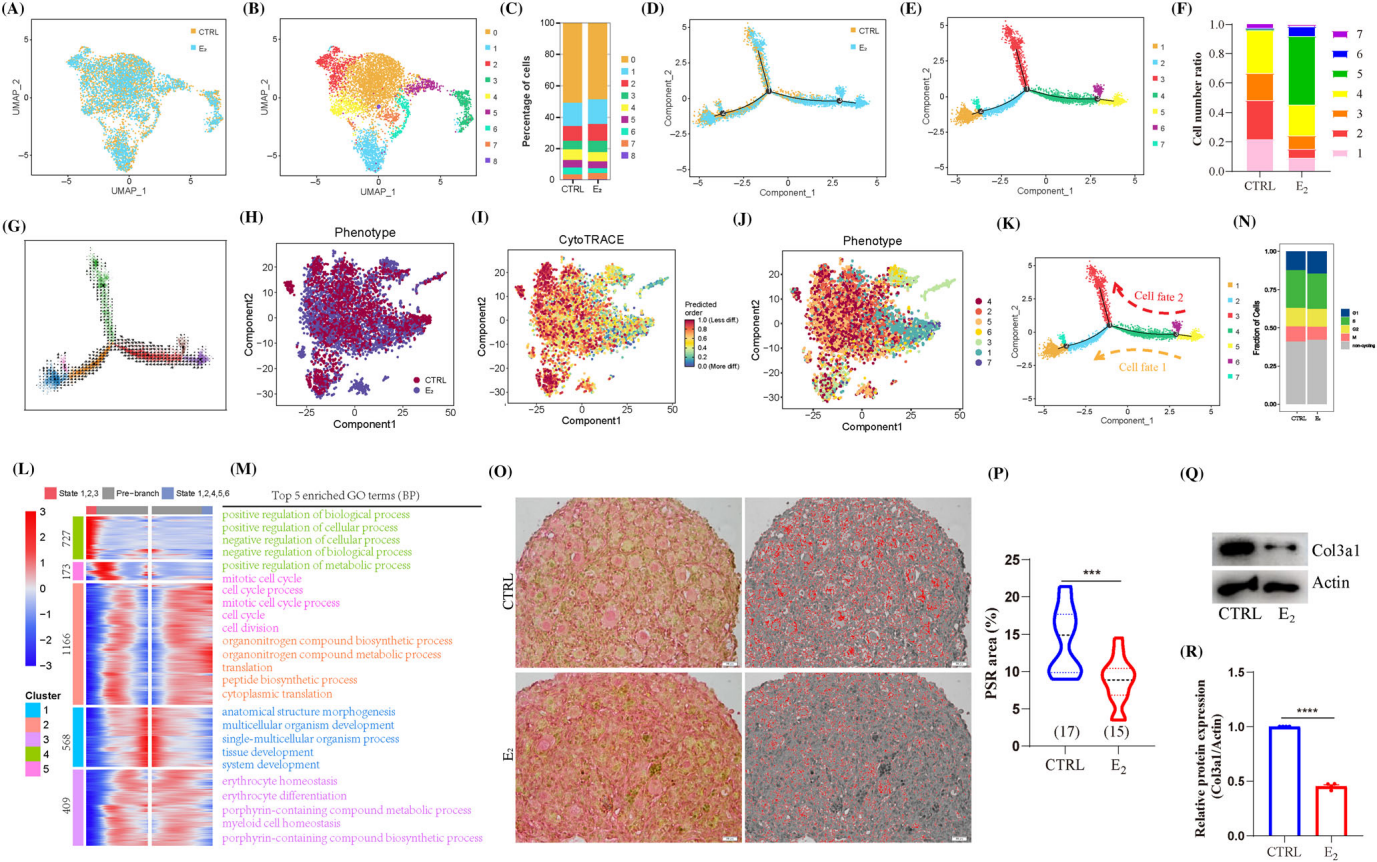
17β‐Estradiol (E_2_) treatment impacted cell development and proliferation of stromal cells, and disordered ovarian microenvironment. (A) Clustering of stromal cells based on uniform manifold approximation and projection (UMAP) coloured by sample groups. (B) UMAP plots of stromal cells based on nine clusters in control (CTRL) and E_2_ groups. (C) Cell number ratio of each cluster in stromal cells. (D) The pseudotime trajectory of stromal cells coloured by sample groups. (E) The pseudotime trajectory of stromal cells based on seven cell states. (F) Cell number ration of seven cell states in the CTRL and E_2_ groups. (G) RNA velocity analysis of stromal cells based on seven cell states. (H–J) CytoTRACE analysis of stromal cells in CTRL and E_2_ groups. (K) Two cell fates of stromal cells in CTRL and E_2_ groups. (L) Pseudotime trajectory of five DEGs sets between two cell fates at branch point one. (M) Top five enriched GO terms (biological processes [BP]) in each gene set. (N) Percentage of cell ratio in stromal cells based on cell cycle analysis in CTRL and E_2_ groups. (O, P) The detection and analysis of Sirius Red staining in ovarian sections between CTRL and E_2_ groups. Scale bar: 20 μm. (Q, R) Detection and analysis of Col3a1 protein in ovaries between CTRL and E_2_ groups. Data are shown as means ± SEM. All experiments were repeated at least three times (***p < 0.001 and ****p < 0.0001).

In the present study, stromal cells (Figure 7A) were also the cell types responding to E_2_ treatment, and subdivided into nine clusters (Figure 7B), the percentage of each cluster is shown in Figure 7C. The top 10 genes in each cluster were analysed (Figure S12A). According to the transcriptome characteristic (Figure S12B), clusters 0, 1, 2, 4, 6 and 8 mainly expressed the characteristic genes of mesenchymal cells (*Col1a2*, *Col1a1*, *Col3a1*, *Dlk1*, *Dcn*, *Mmp2*, *Col6a2*, *Mfap4*, *Sfrp1* and *Igfbp4*); clusters 3 and 5 expressed the relative genes of haemoglobin (*Hba‐a2*, *Hba‐a1*, *Hbb‐bs*, *Hbb‐bt*, *Bpgm*, *Snca*, *Alas2* and *Tent5c*) while cluster 7 expressed the relative genes of mitochondria (*mt‐Nd1/2/4/4L/5*, *mt‐Co1/3*, *mt‐Cytb* and *mt‐Atp6*).

DEGs analysis was applied in stromal cells (Figure S12C), and the number of DEGs was higher than that of other six cell types (Figure S12D; Tables S4–S10). The representative genes (*Cxcl12*, *Stmn1* and *Ltbp2*) were proven by quantitative‐PCR (qPCR) and further supported the alterations after E_2_ treatment (Figure S12E). Concretely, 419 genes were up‐regulated and 2224 genes were down‐regulated after E_2_ treatment (Figure S12C; Table S6). GO enrichment (BP) analysis is shown in Figure S12F, which was mainly related with ‘ribosome,’ ‘Parkinson disease,’ ‘oxidative phosphorylation’ pathways from KEGG analysis (Figure S12G).

For dissecting the fate determination, stromal cells were ordered along a pseudotime trajectory (Figure 7D). Results showed that stromal cells were divided into seven states (Figures 7E and S12H). The representative DEGs of pseudotime are shown in Figure S12I. The DEGs of stromal cells in pseudotime (Figure S12J; Table S21) and the top five enriched GO terms (BP) (Figure S12K) were analysed. According to the cell number ratio (Figure 7F), cell fates 1–4 were mainly contained in the CTRL group (95.98% vs. 45.43%), cell fate 5 was mainly in the E_2_ group, while almost none in the CTRL group (46.57% vs. 0.93%). According to the RNA velocity (Figure 7G) and CytoTRACE analyses (Figure 7H–J) of stromal cells, results showed that state5 was the root of stromal cells and differentiated to state1 and state3 (Figure 7K). Next, we compared the gene expression profiles in two cell fates and observed five gene sets (Figure 7L; Table S13), showing the top five enriched GO terms (BP) (Figure 7M). Altogether, E_2_ treatment impacted the development and disordered cell fates of stromal cells.

### 2.9 E_2_ treatment inhibited the proliferation of stromal cells and altered ovarian microenvironment

The cell cycle was analysed in stromal cells, and the up‐regulated gene expression distribution related to the cell cycle was presented with a bubble map (Figure S13A). Results showed that E_2_ treatment inhibited the proliferation of stromal cells (Figure 7N), presently, the cell proportion in the G2 phase (12.31% vs. 11.72%), S phase (24.76% vs. 23.20%) and M phase (9.90% vs. 8.45%) were decreased, and increased in the G1 phase (11.88% vs. 14.27%) and non‐cycling phase (41.15% vs. 42.36%). Moreover, DEGs of stromal cells showed that *Mki67*, *Top2a*, *Cdk6*, *Ccnd3*, *Cdk2* and *Cdkn1b* were significantly decreased in E_2_ group (Figure S13B). Moreover, collagen content was altered in stromal cells from DEGs analysis after E_2_ treatment (Figure S13C). Presently, the down‐expression of *Col3a1*, *Col4a4*, *Col4a5*, *Col5a2*, *Col6a5*, *Col6a6*, *Col8a2*, *Col9a2*, *Col11a1*, *Col12a1*, *Col15a1*, *Col23a1*, *Col25a1*, and the up‐expression of *Col5a1*, *Col6a3* and *Col6a4*.

Ovarian section staining showed that collagen was significantly decreased in 4 dpp ovaries after E_2_ treatment (Figure 7O,P). Col3a1 expression was decreased (Figure 7Q,R) rather than Col1a1 (Figure S13D,E) in E_2_‐treated ovaries, and the trend was still observed in 21 dpp ovaries (Figure S13F–I). Moreover, the DEGs of PG cells and stromal cells were further analysed, and showed that 1099 DEGs were shared in CTRL and E_2_ groups (Figure S14A), GO terms (BP) (Figure S14B) mainly focused on ‘cellular component organisation or biogenesis,’ ‘macromolecular complex subunit organisation,’ ‘cellular component organisation,’ which involved ‘ribosome,’ ‘oxidative phosphorylation’ (Figure S14C). The GSEA analysis of the oxidative phosphorylation pathway was enhanced in PG cells (Figure S14D) and stromal cells (Figure S14E). In general, E_2_ treatment decreased the capacity for cell proliferation and disordered the ovarian microenvironment.

### 2.10 E_2_ treatment disordered the intercellular communication and key pathways related to PF formation

**FIGURE 8:**
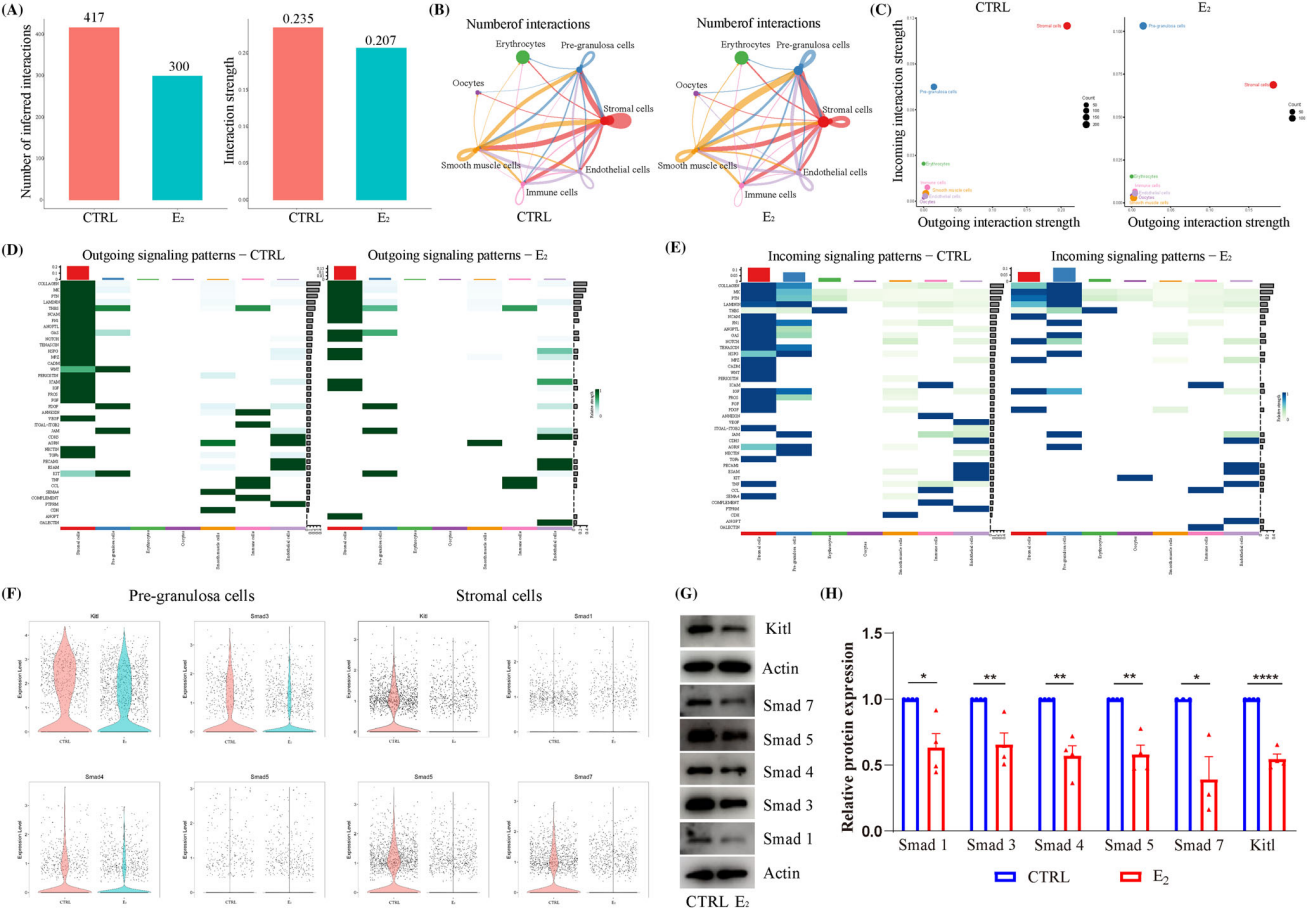
17β‐Estradiol (E_2_) treatment disrupted the intercellular communication and key pathways related to primordial follicle formation. (A) The number of inferred interactions (left) and the interaction strength (right) in 4 dpp ovaries between control (CTRL) and E_2_ groups, respectively. (B) The number of interactions of seven cell types in CTRL (left) and E_2_ (right) groups. (C) The circle plot of the number of interactions among seven ovarian cell types in CTRL (left) and E_2_ (right) groups. (D) The outing signalling patterns of the seven ovarian cell types in the CTRL (left) and E_2_ (right) groups. (E) The incoming signalling patterns of the seven cell types in CTRL (left) and E_2_ (right) groups. (F) Violin diagram of key different expression genes expression in pre‐granulosa cells (left) and stromal cells (right) from single‐cell RNA sequencing. (G, H) The detection and analyses of Kitl, Smad 1, Smad 3, Smad 4, Smad 5 and Smad 7 protein expression in ovaries. Data are shown as means ± SEM. All experiments were repeated at least three times (*p < 0.05, **p < 0.01 and ****p < 0.0001).

The CellChat analysis was performed to analyse the effect of intercellular communication after E_2_ treatment. As shown in Figure 8A, the number of inferred interactions in the CTRL and E_2_ groups was 417 and 300, and the strengths were 0.235 and 0.207, respectively, which showed that E_2_ treatment decreased the intercellular interactions and strengths between oocytes and somatic cells. Notably, stromal cells were the cell type with the strongest incoming and outgoing interaction strength in the CTRL group, but they were disordered after E_2_ treatment, especially, when the strong incoming interaction strength was changed into PG cells (Figure 8B,C).

The overall signalling patterns of the two groups are shown in Figure S14F, and the relative information flow and information flow were presented in Figure S14G,H. Comparing with the outgoing signalling patterns of stromal cells in the CTRL group (Figure 8D), the ANGPTL, TENASCIN, Cell Adhesion Molecules (CADM), WNT, PERIOSTIN, PROS, Fibroblast Growth Factor (FGF), Vascular endothelial growth factor (VEGF), NECTIN, transforming growth factor‐beta (TGFb) and KIT signalling pathways were reduced in the E_2_ group. Except for the changes in thrombospondin (THBS), GAS and WNT signalling, the outgoing signalling patterns in PG cells were almost unchanged. Comparing with the incoming signalling patterns of stromal cells in the CTRL group (Figure 8E), COLLAGEN, PTN, LAMININ, FN1, ANGPTL, growth arrest specific protein (GAS), TENASCIN, HSPG, CADM, WNT, PERIOSTIN, PROS, FGF, ITGAL‐ITGB2, AGRN, TGF‐beta, TNF and SEMA4 signalling pathways were down‐regulated in E_2_ group. Comparing with the incoming signalling patterns of PG cells in CTRL group, the patterns were up‐regulated in E_2_ group, especially COLLAGEN, Midkine (MK), PTN, FN1 and GAS signalling pathways. DEGs analysis showed that *Kitl*, *Smad1*, *Smad3*, *Smad4*, *Smad5* and *Smad7* were decreased in PG cells or stromal cells after E_2_ treatment (Figure 8F). Considering the Kitl signalling and Smad family play vital roles during PF formation,^7^, ^45^, ^49^ Western blotting analysis showed that the expression of Kitl and Smad1/3/4/5/7 were significantly decreased after E_2_ treatment (Figure 8G,H). Moreover, GSEA analysis showed that key pathways related to PF formation were disordered in PG cells and stromal cells after E_2_ supplementation (Figure S14I). Collectively, intercellular communication and key pathways were disordered after E_2_ supplementation in neonatal mice.

## 3 DISCUSSION

Herein, this study defines the cellular mapping and transcriptional alternations associated with E_2_ suppression of PF formation after administering super‐physiological doses daily in 1 dpp, as well as a valuable resource for future exploration by scRNA‐seq in 4 dpp ovaries. After quality control, 21,191 cells were left and identified into seven cell types, including oocytes, PG cells, stromal cells, erythrocytes, immune cells, endothelial cells and smooth muscle cells, which further supported the cell types of the ovary.^45^

Estrogen regulates CBD and PF formation through multiple pathways in the mouse ovary.^29^ Here, E_2_ supplementation significantly decreased the number of PFs and supported the negative role of estrogen during PF formation^8^, ^29^, ^33^; further affected follicle development and resulted in a high incidence of MOFs, and MOFs are correlated with the reduction of fertility and embryonic survival rates in rodents and other wildlife.^37^, ^50^ E_2_ treatment also decreased the capacity of cell proliferation. It is worth mentioning that the ultimate size of the ovarian reserve depends on the appropriate differentiation and proliferation of ovarian support cells.^4^ Although ovaries contain estrogen receptor, the cellular localization has not been described clearly.^29^ Herein, *Esr1*, *Esr2* and *Gper1* were expressed and localised in oocytes, PG cells and stromal cells, and the expression abundance was different in the three cell types, which filled in the cell localization of Estrogen receptors (ERs) during PF formation. Moreover, differences in the expression and location of ERs might explain why knocking out one or both receptors alone has a limited effect on PF formation.^39^, ^40^, ^41^

In oocytes, E_2_ treatment increased key gene expression (*Lhx8*, *Figla* and *Sohlh1*), which was unlike the down‐regulation of these key genes caused by estrogenic pollutant.^46^, ^47^, ^51^, ^52^, ^53^ In *Figla* null female, PFs cannot be formed and oocytes disappear after birth, but there is no significant change in genes associated with ovarian growth and development^54^; in *Sohlh1* knockout mice, although there is no effect on PF formation, primary and growing follicles could not be formed,^55^ and the *Lhx8* knocked out mice are similar to the *Sohlh1* knocked out mice.^55^ Moreover, these genes are marked as early‐follicle,^45^, ^46^, ^47^ and the gene‐expression dynamics in neonatal mice are gradually declined,^56^ while *Ooep* and *Padi6* are up‐regulated in the late‐follicle stage.^45^ E_2_ treatment inhibited oocyte development and was further reinforced by CytoTRACE, so the alteration of these genes became reasonable for the delayed development, which was further supported by the down‐regulation of *Ooep* and *Padi6*. Interestingly, the interaction and cooperation of *Figla*, *Sohlh1* and *Lhx8* play a vital role as multifunctional regulators for oocyte maintenance and differentiation,^57^ so it is possible that E_2_ treatment affects oocyte development via these genes. The diameter of oocytes in the E_2_ group was significantly decreased; similar changes are observed in wild‐type mice with E_2_ treatment, and it is reversed in aromatase knockout mice while the tread is reduced after E_2_ treatment.^34^

In oocytes, oxidative phosphorylation signalling and mitochondrial function were enhanced after E_2_ treatment. In previous research, E_2_ treatment can increase the expression of Complex 1 β subunit 8, Complex IV (COX) or ATP synthase.^58^, ^59^ Herein, some subunits of ETC were significantly up‐regulated in oocytes after E_2_ treatment, such as Ndufb3 and Sdhb, which showed that E_2_ treatment can regulate mitochondrial energy production. The major source of ROS is mitochondria and generated as by‐products of mitochondrial oxidative metabolism.^60^ However, the ROS level of oocytes decreased after E_2_ treatment and maybe due to the alternation of antioxidant capacity (*Gpx1*, *Sod2*, *Prdx2*, *Prdx4* and *Prdx6*), which suggests that estrogen can regulate oxidative stress. Notably, the low level of oxidative phosphorylation is intentional in the early stage of oocytes.^61^ Oocytes maintain ROS‐free mitochondrial metabolism by suppressing Complex I in human and *Xenopus*,^61^ while the related genes of complex I (*Ndufa1/2/3/4/5/6/7/8/9/10/11*, *Ndufb1/2/3/4/5/6/8/10*, *Ndufc2*, *Nduf3/5/6*, *Ndufv1/2/3* and *Uqcrc1/2*) were enhanced after E_2_ treatment. In primate ovarian, the ‘ATP metabolic processes’ and ‘ETC’ are enriched during PF formation, which highly express the mitochondria‐related genes (representative genes *mt‐Nd2* and *mt‐Nd4*) and decrease as follicle development.^23^

Especially, the changes of two cell fates were related to mitochondrial function, and the mitochondria‐related genes (*mt‐Nd1/2/3/4/4L/5*, *mt‐Co1/2/3*, *mt‐Cytb* and *mt‐Atp6/8*) were enriched in the transition state and significantly decreased in late state, which showed the dynamic changes of oxidative phosphorylation during PF formation. A similar phenotype is proved in mice with Di phthalate exposed during PF formation, as known as an endocrine‐disrupting chemicals,^46^ and enhanced glycolysis in granulosa cells can promote activation of PFs.^62^ Collectively, the order of energy supply and demand from glycolytic and oxidative phosphorylation sources during PF formation may be an interesting point. Moreover, SCENIC analysis identified *Rbm3*, *Taf7*, *Fos*, *Jund*, *Hdac2* and *Rab14* as transcriptional regulators. During this process, the activity of *Fos* and *Jund* was decreased in the E_2_ group, which may be the key regulons for that estrogen receptor can activate gene expression via interacting with AP‐1.^63^, ^64^ Altogether, these results show that estrogen affects key gene expression and the development of oocytes, yet it remains unclear about the relative mechanisms.

As a component of PF, the development of PG cells is complex; two distinct pathways of PG cell differentiation support PF formation in mice.^13^ So far, there is few research about the correlation between estrogen and PG cells.^8^, ^29^, ^31^, ^32^, ^33^, ^34^, ^65^, ^66^ Herein, PG cells were identified as EPG or BPG cells according to their characteristic genes.^13^ The RNA velocity analysis firstly proved that EPG cells were recruited into BPG cells in single‐cell level. In a physiological situation, Foxl2‐positive PG cells wrap around the oocyte to form PF.^12^, ^14^, ^17^ Herein, Foxl2 expression in neonatal mice gradually increases during PF formation, but E_2_ treatment decreased Foxl2 expression in 4 dpp ovary and even in 21 dpp ovary. Moreover, E_2_ treatment significantly decreased the recruitment of EPG cells into BPG cells and also reduced the characteristic maintenance of BPG cells (*Foxl2*, *Aard*, *Hsd3b1* and *Serpine2*) while strengthening the characteristics of EPG cells (*Gng13*, *Aldh1a2* and *Krt19*).

The detection of *Foxl2* does not affect PF formation but results in the failure of GC cells differentiation from flat to cubic,^67^ while *Lgr5*‐expressing cell ablation impairs the second wave follicle formation^13^; Aard expresses in Sertoli cells of developing testis,^68^ and may maintain a female hormonal environment and conducive to primary follicle development; Gng13, a gene known to be only expressed at the ovarian surface during sexual differentiation^69^; Aldh1a2 can encode retinoic acid (RA) synthase, which stimulates gonadal cells to produce Foxl2, Esr2 and Wnt4,^70^ while these genes were down‐regulated in PG cells unless Wnt4 after E_2_ treatment. Therefore, the relationship between estrogen and these genes still needs to be further explored. Moreover, E_2_ treatment decreased the capacity of cell proliferation in PG cells. Taken together, E_2_ treatment inhibited cell proliferation, disordered the recruitment and maintenance of two types of PG cells, and resulted in an inappropriate number of EPG and BPG cells, which elucidated the role of estrogen in PG cells during PF formation.

Historically, the relative studies have been mainly focused on oocytes and PG cells,^45^, ^46^, ^47^, ^51^ seriously ignoring other somatic cells. Especially, ovarian stroma has become an exciting frontier,^20^ and a choice against female reproductive ageing with ovarian mesenchymal cells.^19^ Ovarian stroma consists of a mixture of incompletly characterised cells, which have been defined as stromal cells or interstitial cells.^20^ Previous studies used *Nr2f2*, *Tcf21*, *Mfap4*, *Col1a1* and *Dcn* as markers of stromal cells,^13^, ^45^, ^46^ which were also identified in this study. So far, there is few research related to stromal cells during PF formation as well as the relationship with estrogen.^29^, ^31^, ^32^, ^33^, ^34^, ^45^, ^46^, ^47^ Herein, stromal cells were one of the main types of response treatment. E_2_ treatment decreased the capacity of cell proliferation and the proportion of M‐phased cells, which showed that E_2_ treatment inhibited cell proliferation. Monocle analysis showed that E_2_ treatment altered the cell fate of stromal cells, especially cell state5. RNA velocity analysis showed that cell state5 was the initiation site of differentiation; lower expression of *Col4a1* and *Hsd3b1* in state5 and highly expressed genes related to stroma progenitor in pseudotime further supported the site.^71^ Taken together, E_2_ treatment inhibited cell proliferation and development, disordered cell states and fates in stromal cells.

As we know, fibroblasts secrete ECM protein, such as collagen, for cell support, scaffolding and repair.^24^ The female reproductive lifespan can be extended by removing fibrotic collagen from the mouse ovary.^72^ In Esr2‐Null mice ovary,^73^ the expression of ECM is disrupted, such as up‐regulating the expression of Col11a1 and Nidogen2 (Nid2). Herein, the expression of ECM was altered after E_2_ treatment. Presently, down‐regulated the expression of collagen III (*Col3a1*), collagen IV (*Col4a4*, *Col4a5*), collagen V (*Col5a2*), collagen VI (*Col6a5*, *Col6a6*), collagen VIII (*Col8a2*), collagen XI (*Col11a1*), collagen XII (*Col12a1*), collagen XV (*Col15a1*), collagen XXIII (*Col23a1*) and collagen XXV (*Col25a1*), as well as *Nid2* and others; up‐regulated collagen V (*Col5a1*), collagen VI (*Col6a3*) and collagen VI (*Col6a4*). Overall, collagen content decreased in 4 dpp ovaries and even to 21 dpp ovaries after E_2_ treatment. Especially, the relative research is mainly concerned with collagen I and III.^72^, ^74^ E_2_ treatment reduced the expression of Col3al protein rather than Col1a1 in 4 dpp ovaries, and even to 21 dpp ovaries. In rodents and other wildlife, MOFs are correlated with the reduction of fertility and embryonic survival rates,^37^, ^50^ so the inadequate content and altered composition of collagen or ECM may lead to ovulatory disorder and follicular development. In a word, the functional changes of stromal cells impaired the ovarian microenvironment after E_2_ treatment, failing to provide suitable scaffolds for PF formation.

Ovary is a complex organic whole, oocytes communicate with surrounding somatic cells to form PFs.^9^, ^10^ In some estrogen‐like studies involved in intercellular communication between oocytes and PG cells,^47^ which ignore the importance of other cells. In this study, the CellChat analysis showed that not only oocytes communicated with somatic cells frequently, but somatic cells also communicated frequently with each other. However, E_2_ treatment disordered intercellular communication and presently reduced the number and frequency of communication. Moreover, stromal cells were the most powerful inputing and outputing cell type in the 4 dpp ovary. After E_2_ treatment, the strongest incoming interaction strength was changed into PG cells, involving the transformation such as collagen, MK, PTN and others. Specifically, the collagen of incoming signalling pattern was decreased in stromal cells after E_2_ treatment, which showed the indispensable role of the ECM in intercellular communication during PF formation. Moreover, basement membrane plays an essential role in follicle development and the constitute of basement membrane was altered after E_2_ treatment, such as Nid2, as a highly conserved basement membrane glycoprotein.^73^ Collectively, the irreplaceable role of stromal cells is worth exploring, not only in relation to estrogen, but also in understanding the biological process of follicular formation and development under physiological conditions.

Previous studies have confirmed that members of the TGF‐β family and Kit/Kitl signalling were important components of oocyte‐granulosa interactions and joined in PF formation,^45^, ^49^ and play vital roles in many physiological processes, including cell growth, proliferation, migration, adhesion, cell fate determination and differentiation.^10^, ^75^, ^76^ In this study, E_2_ treatment down‐regulated the expression of Smad1/3/4/5/7, and significantly down‐regulated the TGF‐β pathway. Interestingly, liver fibrosis can be attenuated with tyrosine kinase receptor B by inhibited TGF‐β/Smad signalling,^74^ the collagen expression may be inhibited via this pathway for TGF‐β well‐known as a profibrotic cytokine,^74^ as well as the disorder of intercellular communication, development and migration.^24^ Kitl treatment promotes CBD and PF formation in vitro, and inhibition of the Kit/Kitl interaction decreased these processes.^49^, ^77^ Furthermore, Kitl can stimulate oocyte growth.^78^, ^79^ The ovaries culture with Kitl increases ovarian reserve,^80^ and the phosphoinositide 3 kinase (PI3K) pathway might be the primary mediator.^77^ In this study, Kitl expression was significantly decreased and the PI3K‐Akt pathway was inhibited in PG cells and stromal cells in the E_2_ group, which showed the E_2_ treatment might suppress its expression to inhibit PF formation and oocyte growth. Moreover, mitogen‐activated protein kinase (MAPK) signalling, forkhead box O (Foxo) signalling, Wnt signalling, Notch signalling and Hippo signalling also join in PF formation,^4^, ^10^, ^45^, ^81^ and were altered to varying degrees in PG cells and stromal cells after E_2_ treatment, which reflected the broad scope of estrogen action and complexity of regulation. Specially, the differentiation of oocytes and somatic cells must be synchronised to ensure the normal formation of PFs as ovarian development,^9^ yet E_2_ treatment disordered the developmental synchronisation among the three cell types.

**FIGURE 9:**
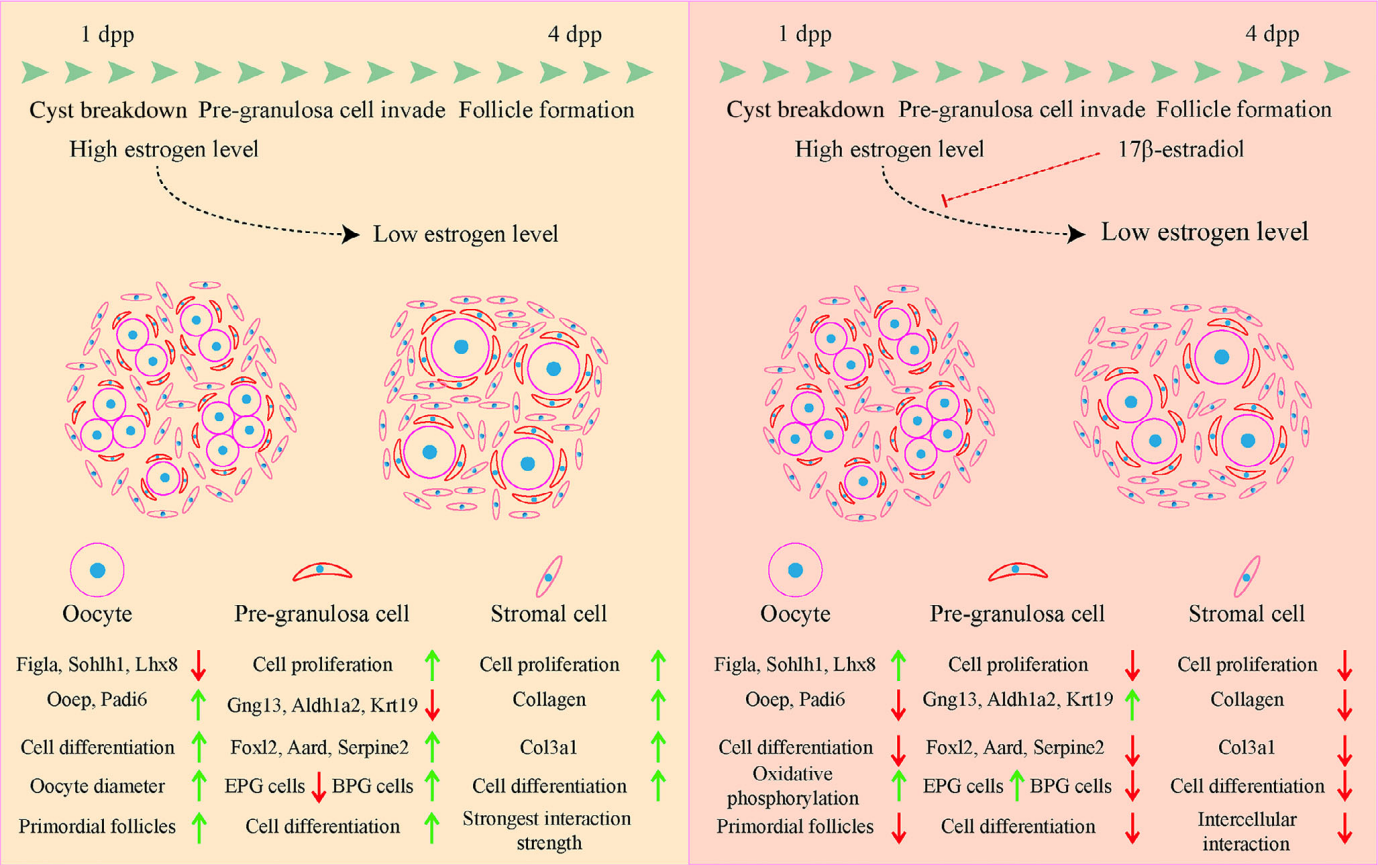
The transcriptional dynamics of 17β‐estradiol suppressing primordial follicle (PF) formation in neonatal mice. The top line marks the time window for PF formation from 1 to 4 dpp. PF formation undergoes cyst breakdown, pre‐granulosa (PG) cell invade and a significant decrease in estrogen level. The left panel indicates normal PF formation in oocytes, PG cells and stromal cells without E_2_ treatment. The right panel displays PF formation suppressed by E_2_ treatment. In oocytes, 17β‐estradiol treatment up‐regulates the key genes expression (*Figla*, *Sohlh1* and *Lhx8*) and enhances oxidative phosphorylation, down‐regulates *Ooep* and *Padi6* expression, inhibits cell differentiation and decreases the number of PFs. In PG cells, 17β‐estradiol treatment inhibits cell proliferation and differentiation, down‐regulates the characteristic genes (*Foxl2*, *Aard* and *Serpine2*) of bipotential pre‐granulosa (BPG) cells and up‐regulates the characteristic genes (*Gng13*, *Aldh1a2* and *Krt19*) of epithelial pre‐granulosa (EPG) cells, decreasing the number of BPG cells while increasing the number of EPG cells. In stromal cells, 17β‐estradiol treatment inhibits cell proliferation and differentiation, down‐regulates the expression of collagen and Cola3a1, and down‐regulates the intercellular interaction.

In summary, this study highlights the alterations in transcriptional dynamics in oocytes, PG cells and stromal cells following E_2_ treatment in neonatal mouse (Figure 9). Specifically, E_2_ treatment influenced key gene expression, mitochondrial function of oocytes, the recruitment and maintenance of PG cells, the cell proliferation of somatic cells, as well as disordered the ovarian microenvironment. These alterations disrupt intercellular communication and the synchronisation of cell development, and ultimately the PF formation is suppressed. Collectively, this study enriches our understanding of the role of estrogen during follicle formation and may contribute to the elucidation of the mechanisms underlying low fertility and embryonic survival‐related disease in humans.

## 4 MATERIALS AND METHODS

### 4.1 Animals

The experiments in this study were reviewed and approved by the Institutional Animal Care and Use Committee of the College of Veterinary Medicine, Northwest A&F University (No. 2021070712). The mice were C57BL/6J mice purchased from the Experimental Animal Center of Xi'an Jiaotong University (Xi'an, Shaanxi, China), bred in a constant‐temperature (22–25°C), fixed‐light environment (12 h light/dark cycle), adequate feed and water. Females were mated with males at a ratio of 1:1; the females with a vaginal plug in the next morning were considered to be E0.5, and delivered pups designated at E19.5 as 1 dpp.

### 4.2 E_2_ treatment

E_2_ (Sigma, E2758) was dissolved at 20 mg/mL in Dimethyl sulfoxide (DMSO) (Sigma, D4540) and stored at −20°C. Before subcutaneous injection administration, 20 μg E_2_ was dissolved in 20 μL corn oil (Solarbio, C7030). Female pups at 1 dpp were continuously administered 20 μg E_2_ treatment for 3 days and were termed the E_2_ group. The mice that were treated without E_2_ were called the CTRL group. The injection doses were determined based on the previous reports.^32^, ^65^, ^66^

### 4.3 Section selection and follicle counting

Ovaries were fixed overnight at 4°C with 10% paraformaldehyde (Solarbio, P1110), embedded in paraffin, and serially sliced into 5‐μm‐thick sections. The sections were selected from around the maximum cross‐sectional area of each ovary, unless otherwise specified. To estimate the total oocytes number, number of oocytes in the cyst and PF, one section in every fifth section was counted. Only oocyte with a visible nucleus was counted and the sum was multiplied by five, as in the previous study.^82^

### 4.4 Immunostaining of ovarian sections

The sections were deparaffinised with xylene and hydrated with different concentrations of ethanol. For haematoxylin and eosin staining, the sections were stained with according to manufacturer's instruction (Solarbio, G1120); for immunohistochemistry, the sections were stained according to manufacturer's instruction (Solarbio, SP0041); for IF staining, the sections were placed in 1× antigen retrieval (Solarbio, C1032) at 96°C for 15 min, blocked with 10% donkey serum for 1 h, then incubated with primary antibody overnight at 4°C. Sections were then incubated with secondary antibody for 2 h. DAPI (Beyotime, C1002) was used to stain the nucleus. The antibodies were presented in Table S1.

### 4.5 TdT‐mediated dUTP Nick‐End Labeling (TUNEL) assay

The sections were detected using a One‐Step TUNEL Apoptosis Assay Kit (Beyotime, C1090). According to the instruction, the slides were treated with proteinase K for 10 min. After washing three times with Phosphate buffered solution (PBS), the slides were incubated at 37°C for 1 h with prepared detection (TdT:Cyanine3 = 1:9). Then cell nuclei were stained and observed under laser‐scanning confocal microscopy (LEICA TCS SP8, Germany).

### 4.6 Single cell library preparation and sequencing

The fresh ovarian tissues (*n* = 6) were cut into pieces, digested with 0.25% trypsin (Hyclone, SV30031.01) and collagenase (2 mg/mL, Sigma, C5138) for 6–8 min at 37°C. Subsequently, the mixture was filtered using a 40 μm cell strainer (BD Falcon, 352340) and washed three times with PBS containing 0.04% bovine serum albumin (Sigma, A1933). The cell viability was above 90% after staining with 0.4% trypan blue, and met the required standards. These cells were labelled with barcodes and subsequently mixed with reverse transcriptase into GEMs. Complementary DNA (cDNA) library was amplified with polymerase chain reaction with sequencing primers. The cDNA libraries were pooled on the Illumina platform (10x Genomics, Illumina). The single cell 3′ protocol produced Illumina‐ready sequencing libraries.

### 4.7 Processing the scRNA‐seq data

The fastq files were processed by CellRanger (v.5.0.0) to obtain a barcode table and gene expression matrix. STAR (v.2.7.2a) used the mouse reference genome GRCm39 in National Center for Biotechnology Information (NCBI) to construct the standard output files required for downstream Seurat. The R package Seurat (v.3.1.1) was used for gene and cell filtration, normalisation, principal component analysis (PCA), variable gene finding and clustering analysis. DoubletFinder (v.2.0.3) was used to remove the effect of double or multiple cells. For quality control, each cell satisfied ‘gene counts between 200 and 4900, Unique molecular identifier (UMI) counts <38,000, and a percentage of mitochondrial genes <25%’ and was retained for downstream analysis. The LogNormalize method was used to normalise the gene expression measurement for each cell according to the formula ‘Log(1 + (UMI A/UMI total) × 10^4^).’ After data integration and scaling with Harmony, PCA was applied and appropriate principal components were selected for the subsequent analysis.

### 4.8 Up‐regulated genes and DEGs analyses

The up‐regulated genes were defined as ‘the gene was expressed in more than 25% of the target cell types, *p* value ≤0.01, log_2_ Fold change (FC) ≥ 0.36.’ DEGs analysis was defined as ‘|log_2_ FC| ≥ 0.36, *p* value <0.05 and the percentage of cells where the gene was detected in specific cluster is more than 10%.’ The GO and KEGG analyses were performed with DEGs. The enrichment of pathway was performed with GSEA, according to the previous report.^83^

### 4.9 Trajectory and RNA velocity analyses

The trajectory analysis of a single cell was performed with Monocle 2 (v.2.10.1) as previously described,^84^ the pseudotime‐dependent gene expression changes were identified by scEpath. Based on the spliced and unspliced transcript reads, the RNA velocity of a single cell was calculated and analysed using scVelo (v.0.2.5).^85^ The velocity fields were projected onto the pseudotime space produced by Monocle 2.

### 4.10 SCENIC analysis

SCENIC R package (v.1.2.4) was performed to identify oocyte‐specific gene regulatory networks, as reported in the previous report.^86^ A log‐normalised expression matrix generated was used as input using Seurat. The gene co‐expression network was identified by GENIE3 (v.1.16.0). Regulons were identified using RcisTarget (v.1.14.0). The AUCell R package (v.1.16.0) was used to determine the activity of each regulon for single cell via the area under curve (AUC) scores.

### 4.11 Cell cycle analysis

The ‘Cellcycle Score’ function in Seurat R package was used to assign cell cycle score by ‘AddModuleScore,’ according to marker genes of cell cycle phase.^87^ Cells with the highest score less than zero were identified as non‐cycling cells; otherwise, it is considered to be the cell cycle.

### 4.12 Cellular communication analysis

To examine cell–cell communication among different cell types, the CellChat packet in R software (v.1.6.1) was used to infer the cellular interaction network as described in the previous report.^88^ The expression matrices after standardisation of CTRL‐ and E_2_‐derived cells were extracted from the Seurat objects, then merged by upgrading the CellChat objects after their creation, followed by comparative analysis.

### 4.13 RNA fluorescence in situ hybridization

The RNA Fluorescence in Situ Hybridization (FISH) assay was performed using the RNA FISH kit (Shanghai GenePharma Co., Ltd., Shanghai, China) according to the manufacturer's instructions. Briefly, xylene was dewaxed, digested by protease K, denatured at 78°C, hybridised with the probe at 37°C and nuclei stained with DAPI. Images were acquired by LEICA (LEICA TCS SP8, Germany). Sequence of the Lgr5 probe was 5′G + TCAGTGT+TCTTAGT+TCAGGCAAA3′ with 5′CY3 modification.

### 4.14 Transmission electron microscopy

Fresh ovaries were fixed with 3% glutaraldehyde, then postfixed with 1% osmium tetroxide. According to the standard TEM procedures, the ovaries were embedded in resin. Serial sectioning with an EM UC7 ultramicrotome (60 nm) was conducted, and the ultrathin sections were stained with lead citrate and uranyl acetate for follow‐up observation with the JEM‐1400‐FLASH Transmission Electron Microscope (JEOL, Japan).

### 4.15 Acetyl‐CoA detection

ACA level was detected using a Mouse Acetyl Coenzyme A ELISA Kit (Jingmei Biological, JM‐1689M2). Briefly, ovarian tissues (*n* = 10) were collected and lysed with RIPA lysis solution. Following the standard requirements of the testing kit, the enzyme‐labelled reagents were incubated at 37°C for 60 min, and the treatment of colour‐developing solution was performed in dark conditions for 15 min and the reaction was terminated. The absorbance value was obtained at the wavelength of 450 nm and the ACA content was calculated.

### 4.16 Citric acid detection

CA level was detected using a CA content detection kit (Solarbio, BC2150). Eight to 10 ovarian tissues were prepared for each test, and the reagents were used following the kit instructions. After incubating at room temperature for 30 min, detected absorbance at the wavelength of 545 nm using a microplate reader.

### 4.17 Oocyte collection and staining

Oocytes were collected with a microscope (OLYMPUS, 7H03989) after digestion as previously described. Briefly, six ovaries were digested and oocytes collected using micromanipulation. For mitochondrial function staining, oocytes were incubated in M2 medium (M7167, Sigma‐Aldrich) with Mito‐Tracker Red (1:2000, Beyotime, C1035), Mito‐Probe JC‐1 Assay Kit (1:200, Beyotime, C2003S) and Reactive Oxygen Species Assay Kit (1:1000, Beyotime, S0033S) at 37°C for 30 min, then washed with M2 medium for three times. The oocytes were then immediately placed on a slide and imaged under a laser‐scanning confocal microscope (LEICA TCS SP8, Germany). The other key genes were stained in a similar way of tissue section staining as in the previous description.

### 4.18 ATP content detection

The ATP content was detected with an enhanced ATP Assay Kit (Beyotime, S0027). Briefly, 100 oocytes and ovaries (*n* = 2) were cleaved with ATP lysate, centrifuged at 12,000 × *g* for 5 min at 4°C, and the supernatant was collected. Samples and standards were transferred into a 96‐well black culture plate after consuming background ATP for 3 min and read with the multi‐mode microplate reader (Tecan Life Sciences). Finally, the ATP content was calculated according to the standard curve.

### 4.19 Collagen content detection

The collagen content of the ovary was detected using a Modified Sirius Red Stain Kit (Solarbio, G1472) according to the instructions. Briefly, sections were selected around the maximum cross section of ovary, conventional dewaxing to water; prepared iron haematoxylin staining solution and stained for 5–10 min, washed with distilled water for 10–20 s, and tap water for 5–10 min, distilled water for three times, 5–10 s each time; then stained with sirius red staining solution for 15–20 min and rinsed slightly with running water; dehydrated by series of ethanol and xylene transparent, finally sealed with neutral gum and captured the images.

### 4.20 RNA extraction and Reverse transcriptase quantitative PCR (RT‐qPCR)

Total RNA was extracted using a MiniBEST Universal RNA Extraction Kit (TaKaRa, 9767), and the cDNA was synthesised using a PrimeScript RT Master Mix reverse transcription kit (TaKaRa, RR036). The qPCR reactions were performed using the SYBR Green Premix Pro Taq HS qPCR Kit (Accurate Biotechnology Co., Ltd, AG11718). The qPCR parameters were as follows: 95°C for 30 s, followed by 40 cycles each at 95°C for 5 s and 60°C for 30 s. The primers for qPCR were listed in Table S2. Unless otherwise stated, the expression was normalised to control values of *GAPDH*, and the level of mRNA quantification was estimated with the 2^−∆∆ct^ method.

### 4.21 Western blotting

The collected ovaries (*n* = 6) were extracted with RIPA lysis solution (Solarbio, R0010) containing protease and phosphatase inhibitor cocktail. Then the samples were mixed with Sodium dodecylsulfate (SDS)‐polyacrylamide gel electrophoresis (SDS‐PAGE) and denatured in boiling water for 10 min.The proteins were separated by SDS‐PAGE and transferred onto a polyvinylidene fluoride membrane. After blocking with 5% no‐fat milk, the primary antibody was incubated at 4°C overnight. Next, the membrane was incubated with the secondary antibody for 2 h after washing three times with Tris Buffered Saline with Tween. Finally, an ECL Plus kit was used for chemiluminescence. The primary and secondary antibodies are shown in Table S1.

### 4.22 Statistical analysis

Results were presented as the mean ± standard error of mean (SEM) and obtained from at least three independent experiments. Statistical analyses were performed with GraphPad Prism software (version 8.0) using an unpaired Student's *t* test for experiments with two groups or a one‐way analysis of variance for experiments with multiple groups. The results with statistically significant differences are indicated by asterisks (*p* < 0.05 denoted by *, *p* < 0.01 denoted by **, *p* < 0.001 denoted by *** and *p* < 0.0001 denoted by ****), *p* > 0.05 defines no significance (ns) and is denoted by ‘ns.’

## AUTHOR CONTRIBUTIONS

Yutong Yan conceived the study, performed major experiments and analyses of data, and writing the manuscript. Hui Zhang, Rui Xu, Linglin Luo, Lu Yin, Hao Wu, Yiqian Zhang, Chan Li, Sihai Lu, Yaju Tang, Xiaoe Zhao, Menghao Pan, Qiang Wei, Sha Peng performed part of the experiments. Baohua Ma took the lead in designing experiments and was in charge of overall direction and planning. All authors read and approved the final manuscript.

## CONFLICT OF INTEREST STATEMENT

The authors declare that they have no competing interests.

## Supporting information


**Data S1.** Supporting Information.

**Data S2.** Supporting Information.

## Data Availability

The data from this study have been deposited in NCBI's Sequence Read Archive (SRA) under the accession number PRJNA1056475.
