# Supplementary material for: Single‐cell sequencing reveals the transcriptional alternations of 17β‐estradiol suppressing primordial follicle formation in neonatal mouse ovaries
Source: Cell Prolif. 2024 Jul 10;57(9):e13713. doi: 10.1111/cpr.13713 (PMC11503257; doi:10.1111/cpr.13713)
Supplement: Supplementary file 1 — Data S1. Supporting Information. [file CPR-57-e13713-s002.docx]

**Supplementary Figures**

**Figure S1. E_2_ treatment inhibited PF formation in neonatal mice.** **(A, B)** The collected ovaries and ovarian area of 4 dpp pups in CTRL and E_2_ groups, respectively. **(C)** The body weight of 4 dpp pups with and without E_2_ treatment. HE staining of sections in 4 dpp ovaries **(D)**, analysis that oocytes within cysts **(E)** and follicles **(F)**, number of oocytes **(G)**. **(H-J)** MVH expression of 4 dpp ovaries in CTRL and E_2_ groups. Data are shown as means ± SEM. All experiments were repeated at least three times. (****p < 0.0001).

**Figure S2. E_2_ treatment inhibited cell proliferation and resulted the incidence of MOFs. (A, B)** The staining and number of TUNEL positive cells in ovarian section between CTRL and E_2_ groups, respectively. **(C, D)** *Casepase3* and *casepase9* expression in 4 dpp ovaries in CTRL and E_2_ groups, respectively. **(E, F)** *Ki67* and *Top2a* expression in 4 dpp ovaries in CTRL and E_2_ groups, respectively. **(G, H)** The detection and analysis of PCNA expression in ovaries between CTRL and E_2_ groups, respectively. **(I)** The IHC staining of IgG and Ki67 in ovarian sections between CTRL and E_2_ groups. The observation of MOFs in 21 dpp **(J)** and 8 weeks (**K**) ovary with and without E_2_ treatment in 1 dpp for three days. **(L, M)** Follicles at different developmental stages were counted between CTRL and E_2_ groups in 21 dpp and 8-week mice, respectively. PF: primordial follicle; PmF: primary follicle; SF: secondary follicle; AF: antral follicle; TO: total oocytes. Data are shown as means ± SEM. All experiments were repeated at least three times. (**p < 0.01, ***p < 0.001, ****p < 0.0001).

**Figure S3. The parameters and quality control of data from scRNA-seq between CTRL and E_2_ groups.** The distribution of basic cell information of each sample before **(A)** and after filtration **(B)** in CTRL and E_2_ groups. **(C)** The relative parameters of scRNA-seq in CTRL and E_2_ groups. **(D)** Scatter plot of the basic information of each sample cell before (Left) and after filtering (Right).

**Figure S4. The analyses of sixteen clusters and cell cycle between CTRL and E_2_ groups.** **(A)** UMAP plots of cells in CTRL and E_2_ groups. **(B)** The percentage of each cluster from CTRL and E_2_ groups. **(C)** The up-regulated genes in each cluster from CTRL and E_2_ groups. **(D**) Heatmap of the top 5 genes in each cluster from CTRL and E_2_ groups. **(E)** Correlation heatmap of each cluster from CTRL and E_2_ groups. **(F-I)** Feature plots of representative marker genes for endothelial cells, smooth muscle cells, erythrocytes, immune cells, respectively. **(J)** Heatmap of genes related to cell cycle in CTRL and E_2_ groups. **(K)** The percentage of cells in different cell cycle phases between CTRL and E_2_ groups.

**Figure S5. The gene expression and difference of oocytes after E_2_ treatment.** **(A)** UMAP plots of seven cell states in oocytes from CTRL and E_2_ groups. **(B)** Heatmap of top 10 genes in each cluster from oocytes sub-population. **(C)** Gene bubble map of marker genes in Pre-, Early- and Late- follicle formation stages in oocytes between CTRL and E_2_ groups. **(D, F)** Violin diagram of *Fos* and *Kit* expression in oocytes from scRNA-seq, respectively. **(E, G)** The level of *Fos* and *Kit* expression in ovaries, respectively. **(H)** The collected oocytes of 4 dpp ovaries in CTRL and E_2_ groups. **(I)** The analysis of oocyte’s diameter in 4 dpp ovaries between CTRL and E_2_ groups. **(J-M)** Violin diagram of *Ooep*, *Figla*, *Sohlh1* and *Lhx8* expression in oocytes from scRNA-seq, respectively. Data are shown as means ± SEM. All experiments were repeated at least three times. (*p < 0.05, **p < 0.01, ****p < 0.0001).

**Figure S6. DEGs analyses of oocytes at early and late follicle stages.** **(A, D)** Volcano plots of DEGs in oocytes at early and late follicle stage between CTRL and E_2_ groups, respectively. **(B, E)** Top 10 GO terms (BP) from DEGs of oocytes at early and late follicle stages, respectively. **(C, F)** Top 10 pathways from DEGs of oocytes at early and late follicle stages, respectively. **(G)** Venn diagram of DEGs of oocytes at early and late follicle stages. **(H)** Top 10 GO terms (BP) of the shared DEGs in oocytes at early and late follicle stages. **(I)** Top 10 pathways of the shared DEGs in oocytes at early and late follicle stages.

**Figure S7. E_2_ treatment disordered cell fates of oocytes and regulated the activity of regulations. (A)** The pseudotime trajectory of oocytes by three cell states. **(B)** Expression of developmental marker genes (*Ppia*, *Sycp3*, *Eif4a1*, *Figla*) in oocytes with pseudotime trajectory. **(C-E)** CytoTRACE analysis for reconstructing cellular differentiation trajectory in oocytes from CTRL and E_2_ groups, cell states. **(F)** Representative genes (*Alas2*, *Ube2l6*, *Prdx2*, *Gpx1*, *Blvrb*, *Malat1*) of the two cell fates in oocytes. **(G)** Heatmap of regulons activity in oocytes analyzed by SCENIC analysis in CTRL and E_2_ groups.

**Figure S8. The expression of characteristic genes in BPG and EPG cells**. **(A)** Violin diagram of characteristic genes expression in BPG and EPG cells. **(B)** The top 10 up-regulated genes in each cluster from PG cells. **(C)** The top 10 up-regulated genes in BPG and EPG cells. **(D)** Expression of *Amhr2* in the PG cells. **(E)** Volcano plots of DEGs in PG cells. **(F)** The top 10 GO terms based on the DEGs of PG cells in CTRL and E_2_ groups. **(G)** The top 10 pathways based on the DEGs of PG cells in CTRL and E_2_ groups.

**Figure S9. Foxl2 expression and DEGs analyses of EPG and BPG cells.** **(A, B)** The detection and analysis of Foxl2 expression in 1 dpp, 2 dpp, 4 dpp ovaries, respectively. **(C, D)** The detection and analysis of Foxl2 expression in 21 dpp ovaries after E_2_ treatment for 3 days in 1 dpp, respectively. **(E, G)** Violin diagram of *Nr5a2* and *Krt79* expression in PG cells from scRNA-seq, respectively. **(F, H)** The level of *Nr5a2* and *Krt79* expression, respectively. **(I, L)** Volcano plots of DEGs in EPG and BPG cells, respectively. **(J, M)** Top 10 GO terms (BP) from DEGs in EPG and BPG cells, respectively. **(K, N)** Top 10 pathways from DEGs in EPG and BPG cells, respectively. **(O)** Venn diagram of DEGs in EPG and BPG cells. **(P-R)** Top 10 GO terms (BP) from shared and different DEGs in EPG and BPG cells. Data are shown as means ± SEM. All experiments were repeated at least three times. (**p < 0.01, ***p < 0.001).

**Figure S10. The analyses of cell cycle and cell fates in PG cells.** **(A)** Heatmap of the expression related to cyclin genes in different cell cycles. **(B)** Bubble map of up-regulated gene expression distribution related to cell cycle. **(C)** Representative DEGs (*Mki67*, *Cdkn1b*, *Ccnd1*) of PG cells related with cell cycle in CTRL and E_2_ groups. **(D)** The pseudotime of PG cells in CTRL and E_2_ groups. **(E)** The distribution of seven cell states of PG cells in CTRL and E_2_ groups. **(F, G)** The expression of characteristic genes related to EPG and BPG cells in pseudotime, respectively.

**Figure S11 The analyses of DEGs of EPG and BPG cells in pseudotime. (A)** Heatmap of DEGs expression of EPG cells in pseudotime. **(B)** Top 5 enriched GO terms (BP) from DEGs of EPG cells in each genes set. **(C)** Heatmap of DEGs of BPG cells in pseudotime. **(D)** Top 5 enriched GO terms (BP) from DEGs of BPG cells in each genes set.

**Figure S12 The analyses of stromal cells after E_2_ treatment. (A)** Heatmap of top 10 genes in each cluster of stromal cells. **(B)** Gene bubble map in each cluster from stromal cells. **(C)** Volcano plot of DEGs in stromal cells from CTRL and E_2_ groups. **(D)** Number of DEGs in six cell types in CTRL and E_2_ groups. **(E)** The violin diagram of DEGs (*Cxcl12*, *Stmn1*, *Ltbp2*) in stromal cells from scRNA-seq and detection by q-PCR, respectively. **(F)** Top 10 GO terms (BP) of DEGs in stromal cells between CTRL and E_2_ groups. **(G)** Top 10 pathways of DEGs in stromal cells. **(H)** The distribution of stromal cell from CTRL and E_2_ groups in pseudotime. **(I)** The representative gene of stromal cells in pseudotime. **(J)** Heatmap of DEGs from stromal cells in pseudotime. **(K)** Top 5 enriched GO terms from DEGs of stromal cells in each genes set.

**Figure S13 E_2_ treatment inhibited the proliferation of stromal cells and disordered ovarian microenvironment**. **(A)** Gene bubble map related to cell cycle in stromal cells. **(B)** Violin diagram of DEGs (*Mki67*, *Top2a*, *Cdkn1b*, *Ccnd3*, *Cdk2*, *Cdk6*) in stromal cells related to cell cycle. **(C)** Heatmap of DEGs in stromal cells related to collagen between CTRL and E_2_ groups. **(D, E)** The detection and analysis of Col1a1 in 4 dpp ovaries from CTRL and E_2_ groups. **(F, G)** The detection and analysis of Col1a1 and Col3a1 in 21 dpp ovaries after E_2_ treatment for three days in 1 dpp, respectively. **(H, I)** Sirius Red staining and analysis of 21 dpp ovaries after E_2_ treatment for three days in 1 dpp mice, respectively. Data are shown as means ± SEM. All experiments were repeated at least three times. (**p < 0.01, ****p < 0.0001).

**Figure S14 E_2_ treatment disordered the intercellular communication and key pathways. (A)** Venn diagram of DEGs between PG cells and stromal cells after E_2_ treatment. **(B, C)** The top 10 GO terms (BP) and pathways from the shared DEGs in PG cells and stromal cells, respectively. **(D, E)** The GESA analysis of oxidative phosphorylation pathway in PG cells and stromal cells, respectively. **(F)** The overall signaling patters of seven cell types in CTRL and E_2_ groups. **(G, H)** Relative information flow and Information flow in CTRL and E_2_ groups. **(I)** The GSEA analysis of key pathways during PF formation in PG cells (Green) and stromal cells (Red).

**Supplementary tables**

Table S1. Primary antibodies for IF and WB.

Table S2. Primer sequences for q-PCR analysis.

Table S3. Up-regulated genes in each cluster from scRNA-seq.

Table S4. The analysis of DEGs in oocytes between CTRL and E_2_ groups.

Table S5. The analysis of DEGs in PG cells between CTRL and E_2_ groups.

Table S6. The analysis of DEGs in stromal cells between CTRL and E_2_ groups.

Table S7. The analysis of DEGs in endothelial cells between CTRL and E_2_ groups.

Table S8. The analysis of DEGs in erythrocytes between CTRL and E_2_ groups.

Table S9. The analysis of DEGs in immune cells between CTRL and E_2_ groups.

Table S10. The analysis of DEGs in smooth muscle cells between CTRL and E_2_ groups.

Table S11. The analysis of DEGs in oocytes according to three states at brance 1.

Table S12. The analysis of DEGs in PG cells according to seven states at brance 3.

Table S13. The analysis of DEGs in stromal cells according to seven states at brance 1.

Table S14. The analysis of DEGs in early oocytes between CTRL and E_2_ groups.

Table S15. The analysis of DEGs in late oocytes between CTRL and E_2_ groups.

Table S16. The analysis of up-regulated genes in PG cells between CTRL and E_2_ groups.

Table S17. The analysis of DEGs in EPG cells between CTRL and E_2_ groups.

Table S18. The analysis of DEGs in BPG cells between CTRL and E_2_ groups.

Table S19. The analysis of DEGs in EPG cells according to pseudotime.

Table S20. The analysis of DEGs in BPG cells according to pseudotime.

Table S21. The analysis of DEGs in stromal cells according to pseudotime.
